# Altered metabolic function induced by Aβ‐oligomers and PSEN1 mutations in iPSC‐derived astrocytes

**DOI:** 10.1111/jnc.16267

**Published:** 2024-12-18

**Authors:** Richard J. Elsworthy, Mattea J. Finelli, Sarah Aqattan, Connor Dunleavy, Marianne King, Adele Ludlam, Marta A. Tarczyluk, Sophie L. Allen, Sophie Prosser, Rui Chen, Sandra Martinez Jarquin, Dong H. Kim, James Brown, H. R. Parri, Sarah Aldred, Eric J. Hill

**Affiliations:** ^1^ School of Sport, Exercise and Rehabilitation Sciences, College of Life and Environmental Sciences University of Birmingham Birmingham UK; ^2^ Biodiscovery Institute, University of Nottingham, School of Medicine University Park Nottingham NG7 2RD Nottingham UK; ^3^ School of Biology, College of Health and Life Sciences Aston University Birmingham UK; ^4^ Sheffield Institute for Translational Neuroscience University of Sheffield Sheffield UK; ^5^ School of Pharmacy, College of Health and Life Sciences Aston University Birmingham UK; ^6^ NIHR Birmingham Biomedical Research Centre University Hospitals Birmingham NHS Foundation Trust, University of Birmingham Birmingham UK; ^7^ Centre for Analytical Bioscience, Advanced Materials & Healthcare Technologies Division, School of Pharmacy University of Nottingham Nottingham UK; ^8^ Department of Chemistry Loughborough University Loughborough UK

**Keywords:** Alzheimer's, astrocytes, gliosis, inflammation, metabolism, stem cells

## Abstract

Altered energy metabolism in Alzheimer's disease (AD) is a major pathological hallmark implicated in the early stages of the disease process. Astrocytes play a central role in brain homeostasis and are implicated in multiple neurodegenerative diseases. Although numerous studies have investigated global changes in brain metabolism, redox status, gene expression and epigenetic markers in AD, the intricate interplay between different metabolic processes, particularly in astrocytes, remains poorly understood. Numerous studies have implicated amyloid‐β and the amyloid‐β precursor in the development and progression of AD. To determine the effects of amyloid‐β peptides or the impact of amyloid‐β precursor protein processing on astrocyte metabolism, we differentiated astrocytes from induced pluripotent stem cells derived from people with early onset familial AD and controls. This study demonstrates that familial AD‐derived astrocytes exhibit significantly more changes in their metabolism including glucose uptake, glutamate uptake and lactate release, with increases in oxidative and glycolytic metabolism compared to acute amyloid‐β exposure. In addition to changes in major metabolic pathways including glutamate, purine and arginine metabolism and the citric acid cycle, we demonstrate evidence of gliosis in familial AD astrocytes highlighting a potential pathological hallmark. This suggests that chronic alterations in metabolism may occur very early in the disease process and present significant risk factors for disease progression for patients with early onset AD. These findings may also reveal important drivers of disease in late onset dementia and highlights key targets for potential diagnostic features and therapeutic agents in the future.

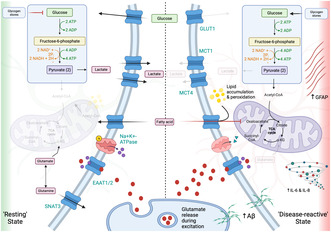

## Graphical abstract

In this study, we investigate how amyloid beta oligomers and familial Alzheimer's disease‐linked Presenilin 1 mutations alter the metabolism of astrocytes derived from induced pluripotent stem cells. Our findings reveal that amyloid beta and Presenilin 1 mutations alter both glucose and glutamate uptake, increase lactate release, and induce reactive gliosis as well as shifts in oxidative and glycolytic metabolism. Overall, the study highlights that these metabolic disruptions may occur early in disease development, highlighting the key role of astrocytes in Alzheimer's disease pathology and suggests that early diagnostic markers and therapeutic targets should address metabolic dysfunction.

**Abbreviations**
AD: Alzheimer's disease
ADAM10: ‘a disintegrin and metalloproteinase’‐10
ADM: astrocyte differentiation medium
ALDH1L1: aldehyde dehydrogenase 1 family member L1
AMG: amyloglucosidase enzyme
AOPI: acridine orange and propidium iodide
ApoE: apolipoprotein E
Aβ: amyloid‐β
CM: conditioned media
DAB: 1,4‐dideoxy‐1,4‐imino‐d‐arabinitol
dbcAMP: dibutyryl cyclic adenosine monophosphate
dPBS: Dulbeco's phosphate‐buffered saline
ECAR: extracellular acidification rate
ELISA: enzyme‐linked immunosorbent assay
fAD: familial Alzheimer's disease
GFAP: glial fibrillary acidic protein
HFIP: hexafluorisopropanol
hNPCs: human neural precursor cells
IL‐6: interleukin‐6
IL‐8: interleukin‐8
iPSC: induced pluripotent stem cell
MTT: 3‐(4,5‐dimethylthiazol‐2‐Yl)‐diphenyltetrazolium bromide
OCR: oxygen consumption rate
PFA: paraformaldehyde
PSEN1: presenilin 1
QC: quality control
RFU: relative fluorescence units
sAβPP‐α: soluble amyloid‐β precursor protein
TBOA: DL‐threo‐benzyloxyaspartic acid

## 1 INTRODUCTION

Despite its relatively small proportion of total body mass (2%), the human brain requires a substantial amount of energy to sustain its complex functions, accounting for approximately 20% of total energy consumption (Attwell & Laughlin, 2001; Dienel, 2019) with neurons consuming the majority (75%–80%) of this energy (Harris et al., 2012; Hyder et al., 2013; Yu et al., 2018). This high energy demand is attributed to the establishment of electrochemical gradients, axonal transport and neurotransmitter recycling (Harris et al., 2012). Glucose is considered the primary energy substrate in the brain, although other metabolites such as ketones and lactate can also be utilised (Cunnane et al., 2020; Magistretti & Allaman, 2015). Astrocytes play crucial roles in neuronal signalling, homeostasis and neuroprotection. They form intricate interactions with neurons, creating a ‘tripartite synapse’ and providing energetic metabolites from blood vessels to distant neurons through gap junctions (Clasadonte et al., 2017; Dermietzel et al., 1991; Rouach et al., 2008). Astrocytes store glycogen, the brain's exclusive glycogen reservoir and rapidly convert it into lactate, which serves as a crucial fuel source for neurons during increased neuronal activity (Brown & Ransom, 2007; Dienel & McKenna, 2014). Moreover, astrocytes exhibit neuroprotective functions by preventing glutamate toxicity and oxidative stress through the generation of antioxidants like glutathione (Ding et al., 2021). Accumulating evidence suggests that Aβ, a key player in Alzheimer's disease (AD) pathology, induces metabolic dysfunction in both neurons and astrocytes, possibly through mechanisms involving oxidative stress, mitochondrial dysfunction, impaired calcium signalling and disruption of essential enzymes (Butterfield et al., 2001; de la Monte & Tong, 2014). Recent evidence of a possible role for astrocytes as the link between Aβ, tau pathology and cognitive symptoms has contributed to the increasing interest in astrocytic proteins as biomarkers of AD progression (Bellaver et al., 2023; De Bastiani et al., 2023).

Disruptions in the uptake of energy substrates by the brain can have severe consequences for normal brain function. Studies utilising fluorodeoxyglucose positron emission tomography have revealed a decrease in glucose uptake prior to the onset of disease symptoms in individuals at risk of developing AD, including those with familial AD (fAD) and apolipoprotein E (ApoE) 4 carriers. (Kyrtata et al., 2021; Mosconi et al., 2008, 2009; Reiman et al., 2004) While neuronal loss, a hallmark of AD, could explain the decline in glucose utilisation, evidence suggests that changes in energy metabolism occur before neuronal loss, potentially contributing to the development and progression of the disease (Reiman et al., 2004; Rouach et al., 2008; Zhang et al., 2023). Although numerous studies have investigated global changes in brain metabolism, redox status, gene expression, and epigenetic markers in AD, the intricate interplay between different metabolic processes, particularly in astrocytes, remains poorly understood. Brain glucose consumption is not homogeneous, and different cell types exhibit distinct glycolytic/aerobic metabolic profiles (Magistretti & Allaman, 2015). Several studies have reported altered glucose metabolism, insulin signalling and response to oxidative stress in astrocytes in AD (Garwood et al., 2017; Simpson et al., 2011). The ApoE ε4 variant (a known risk factor for AD) is associated with significant metabolic changes in astrocytes at the earliest stages of the disease (Lindner et al., 2022; Nam et al., 2023; Zulfiqar et al., 2019). As altered metabolism is recognised as an early event in the development and progression of AD it is essential to understand the role of astrocytes in this process (Allaman et al., 2010; Zilberter et al., 2013).

Induced pluripotent stem cell (iPSC)‐derived models of the AD brain are an increasingly popular tool to investigate cell type‐specific characteristics and cell–cell interactions. These models have demonstrated that processes including, amyloid generation, calcium homeostasis, amino acid metabolism and neuron–astrocyte transmitophagy are altered, with astrocytic cells displaying characteristics of a reactive phenotype (Jones et al., 2017; Lampinen et al., 2022; Oksanen et al., 2017; Salcedo et al., 2021). More recently, both iPSC derived astrocytes from late‐onset and familial AD have been shown to display mitochondrial dysfunction specifically linked to a deficit in the glycolytic enzyme, hexokinase‐1 (Bell et al., 2024).

In this paper, the effect of Aβ oligomers on metabolic function in iPSC‐derived astrocytes was investigated. We demonstrate that exposure to Aβ oligomers induces a significant shift in energy metabolism and elicits a ‘reactive’ state in healthy astrocytes. Building upon this, we characterised astrocytes differentiated from fAD patient‐derived iPSC cell lines carrying Presenilin 1 (PSEN1) mutations. We show that PSEN1 astrocytes generate more Aβ than ‘healthy’ controls and display an altered metabolic status with features of reactive gliosis. Overall, the data presented will allow for a better understanding of the underlying features of astrocyte dysfunction in AD. By focusing on the molecular mechanisms underlying astrocyte dysfunction in AD, novel therapeutic strategies may emerge, providing potential avenues to restore energy metabolism and counteract disease progression.

## 2 MATERIALS AND METHODS

### 2.1 Neuralisation of iPSC's to human neural precursor cells

fAD PSEN1 (R278I) iPSC line was obtained from Prof. Selina Wray (UCL, UK; Arber et al., 2020). The initial tissue was provided under the ethical approval of NHS Research Authority NRES Committee London‐Central (REC# 08/H0718/54 + 5). R278I cells were cultured and monitored for colony size and changes in morphology before undergoing neural induction as described (Elsworthy et al., 2021) based on adapted protocols previously developed (Chambers et al., 2009; Shi et al., 2012). Healthy control (ax0018) and PSEN1 human neural precursor cells (hNPCs) carrying L286V (ax0112) and A246E (ax0114) mutations were purchased from Axol Bioscience (Cambridge, UK). Cell lines from Axol Biosciences are officially certified by HPSreg®. The cell lines used in this study are not listed as commonly misidentified cell lines by the International Cell Line Authentication Committee (ICLAC; http://iclac.org/databases/cross‐contaminations/).

### 2.2 Astrocyte differentiation of human neural stem cells

Healthy control and PSEN1 hNPCs were plated as previously described (Elsworthy et al., 2021) in parallel to the R278I generated hNPCs (see above). Briefly, the control and three fAD PSEN1 cells were seeded at a density of 7 × 10 (Hyder et al., 2013) cells/cm (Dienel, 2019) in neural plating medium (Axol Bioscience, Cambridge, UK) on Matrigel‐matrix (356237, Corning) coated wells. After 24 h cells were washed with D‐PBS before medium was exchanged for astrocyte differentiation medium (ADM; STEMdiff^tm^ Astrocyte differentiation kit #100‐0013, StemCell Technologies, Cambridge, UK) and a full media exchange was completed every day for 7 days. Cells were passaged at around 80% confluency using 1 mL/well Accutase™ (A6964, Merck, UK) and dissociation was stopped with 4 mL/well ADM. Cells were plated at 5 × 10^4^ /cm^2^ density and maintained in a 37°C, 5% CO_2_/ 95% air atmosphere with a total medium exchange every other day, through two subsequent passages, before switching to astrocyte maturation medium (STEMdiff^tm^ Astrocyte maturation kit #100‐0016, StemCell Technologies, Cambridge, UK). Cells were seeded to a density of 2.5 × 10 (Yu et al., 2018)/cm (Dienel, 2019) onto tissue culture plastic on Matrigel‐matrix and media was changed every other day for 7 days or until reaching 80% confluency. Upon reaching 80% confluency and maturation, cells were passaged as before and cultured in astrocyte maintenance media (ScienCell astrocyte media, Cat #1801) until ready for analysis at day 45. Each replicate (*n*) was represented by a separate astrocyte induction process, from which technical repeats were generated. Cells were routinely tested for mycoplasma contamination by luminescence assay (Lonza MycoAlert™ PLUS, LT07‐703). For information on cell lines used, see Table  S1.

#### 2.2.1 Immunocytochemistry

Cells were fixed in 4% (v/v) paraformaldehyde (PFA) in Dulbeco's phosphate buffered saline (dPBS). The cells were then incubated for 10 min in PBS with 0.2% (v/v) Triton X‐100 followed by blocking for 1 h in dPBS containing 0.2% (v/v) Triton X‐100 and 3% (w/v) bovine serum albumin (A9418 Sigma‐Aldrich, UK). Primary antibodies for Aldehyde Dehydrogenase 1 Family Member L1 (ALDH1L1; 702 573, Invitrogen), Glial fibrillary acidic protein (GFAP; 14‐9892‐82, Invitrogen) and S100β (PA5‐78161, Invitrogen) were diluted in blocking buffer and added for 1 h. Following primary antibody incubation, cells were washed with blocking buffer and appropriate secondary antibodies, Alexa Fluor® 488 AffiniPure Goat Anti‐Rabbit IgG (1:2000, 111‐545‐144, Jackson Laboratories) and Alexa Fluor® 633 Goat anti‐Mouse IgG (1:2000, A‐21052, ThermoFisher Scientific) were added for 1 h. Cells were mounted in Prolong™ Gold Antifade Mountant with DAPI (P3935, ThermoFisher Scientific) to glass slides and imaged using an EVOS m5000 imaging system (AMF5000, ThermoFisher, UK).

#### 2.2.2 Preparation and treatments of synthetic Aβ1‐42 oligomers

Human hexafluorisopropanol (HFIP) Aβ1‐42 (AG968, Sigma Aldrich) was prepared in oligomeric form as previously described (Stine et al., 2011). Briefly, human HFIP Aβ1‐42 was resuspended in DMSO to 5 mM. Monomers were diluted in F‐12 culture media, without phenol red, to a concentration of 100 μM and incubated for 24 h at 4°C. Confirmation of oligomerisation and cellular uptake can be seen in Figure S1. To determine the possible toxic effects of human Aβ1‐42 oligomers on the metabolism of astrocytic cells derived from ‘healthy’ patient hNPCs, cells were treated with oligomeric Aβ1‐42 over a range of concentrations based on our previous studies compared to the loading control (DMSO; Tarczyluk et al., 2015). Astrocytes were incubated for either 4 or 48 h at 37°C in a humidified atmosphere of 5% CO2. Subsequently, conditioned media (CM) was collected and centrifuged at speed of 200 *g* for 5 min. Protein lysates were collected from cells using ice cold RIPA buffer (R2078, Sigma Aldrich) with Halt™ 100x protease inhibitor cocktail added (78 440, Thermofisher). CM and lysates were transferred into 1.5 mL sterile microcentrifuge and stored at −80°C for future measurements.

### 2.3 Total cellular protein quantification

The protein concentration of cell lysates was determined using a modified protocol of the bicinchoninic acid protein assay (Smith et al., 1985) to enable standardisation of assays.

#### 2.3.1 MTT assay

iPSC‐derived astrocytes were seeded into 96 well plates at a density of 8 × 10 (Yu et al., 2018) per well. Triplicate technical repeats for each control and experimental condition were used. For the assay, 3‐(4,5‐dimethylthiazol‐2‐Yl)‐diphenyltetrazolium bromide (MTT, CT01‐5, Sigma‐Aldrich, UK) stock solution was diluted in F12 medium without phenol red (1:5), added to each well and incubated for 3 h (37°C). The MTT solution was then aspirated and DMSO (50 μL) was added to each well. Cells were placed on a plate shaker (500 rpm) for 30 s followed by incubation for 10 min (37°C). Finally, absorbance was read at 590 nm (Fluostar Omega, BMG Labtech).

#### 2.3.2 AOPI cell viability count

The viability of astrocytes following treatment with Aβ1‐42 oligomers was measured using an automated dual fluorescence cell count (Cellometer 2000, Nexcelom). Briefly, astrocytes were treated with either 0.2 μM,1 μM or 2 μM Aβ1‐42 and compared to the untreated control for 48 h. Acridine orange (AO) and propidium iodide (PI) fluorescence imaging was quantified to determine live/dead, cell size and total cell counts (ViaStain™ AOPI Staining Solution, Nexcelom).

#### 2.3.3 Seahorse analytics

Oxygen consumption rate (OCR), extracellular acidification rate (ECAR), mitochondrial respiration indicators and glycolysis indicators, respectively, were measured from live cells in real time using Seahorse Extracellular Flux (XF) 24 Analyzer (Agilent Technology). OCR and ECAR were measured simultaneously. Matured astrocytes were plated on Matrigel‐coated XF24 TC plate at a density of 40 000 cells/well in astrocyte maturation medium and cultured overnight. For Aβ treatment comparisons, 0, 0.2, 1 and 2 μM were added to control cells 48 h prior to commencing the assay. Before conducting the assay, media was changed to Seahorse XF Assay medium (Seahorse RPMI supplemented with 2 mM glutamine, 1 mM sodium pyruvate and 10 mM glucose). Oligomycin (2 μg/mL), BAM‐15 (3 μM), antimycin/rotenone (2 μM) and 2‐Deoxyglucose (50 mM) injections were performed in line with manufacturer's instructions using 3 min mix time, 3 min wait time and 3 min measure cycle. Following the assay, results were normalised using CyQUANT nucleic acid stain (C7026, Invitrogen). Data were analysed using Wave program (Agilent Technology).

##### Glycogen assay

Astrocytes cultured in 12 well plates were scraped into 300 μL ice cold HCL (30 mM), sonicated for 15 s before dilution 1:2 with D‐PBS and the diluted samples were mixed with 0.1 M acetate buffer at a pH of 4.6. The diluted sample was divided equally into two separate microfuge tubes, one tube to measure the amount of glycogen and the second tube to measure the free glucose. Next, an equal volume 1 mg/mL amyloglucosidase enzyme (AMG) stock (Sigma‐Aldrich, UK), which was prepared by adding 75 μL of AGM reagent in 1 mL of acetate buffer with a pH of 4.6. Afterwards, all the samples were incubated at 57.5°C on a heat block for 2 h. After incubation, 30 μL of sample was transferred into a 96‐well plate and added 100 μL of Hexokinase enzyme reagent (Sigma‐Aldrich, UK), and then mixed and incubated at room temperature for 15 min (Figures 2 and 3). The absorbance was read at 370 nm using Thermo multiscan EX 96‐well plate reader (Thermofisher, UK). The protein content levels in the cell lysate samples were determined using BCA assay in order to normalise the glycogen values. The ability of iPSC derived astrocytic cells to store and breakdown glycogen was assessed using physiological cues such as hypoglycaemia: 1,4‐dideoxy‐1,4‐imino‐d‐arabinitol (DAB; Sigma‐Aldrich, UK) or drugs including: Dibutyryl cyclic adenosine monophosphate (dbcAMP; Tocris, UK), Isoproterenol (Tocris, UK), Ouabain (Tocris, UK) and DL‐threo‐benzyloxyaspartic acid (TBOA; Tocris, UK).

#### 2.3.4 Glucose assay

Glucose levels in culture media were quantified using a bioluminescent NADH detection assay according to manufacturer's instructions (Glucose‐glo, Promega). Briefly, the addition of glucose detection reagent 1:1 to samples (12.5 μL) in a 384‐well plate was incubated for 1 h or until a stable luminescent signal was achieved. Luminescence was plotted against serially diluted standards from 50 to 0.8 μM to calculate glucose levels in cell conditioned culture media.

#### 2.3.5 Lactate and glutamate assays

Glutamate (ab83389, Abcam) and Lactate (65 331, Abcam) levels were quantified in cell conditioned media by colorimetric assays as per the manufacturer's instructions. For glutamate uptake and subsequent lactate release, a modified protocol from Mahmoud et al. (2019) was utilised. Briefly, astrocytes were seeded in 12‐well plates at 3.5 × 10^4^ and cultured for 24 h before treatment with Aβ for 48 h. Cells were washed two times with Hank's Balanced Salt Solution (HBSS, Gibco™ 14025092) containing Ca^2+^ before incubation for 4 h (37°C, 5% CO_2_) with 200 μM glutamate. Glutamate uptake by astrocytes was measured by subtracting the amount of glutamate measured in the CM from the amount added to the cells. The protein content levels in the cell lysate samples were also determined using BCA assay to normalise values (Mahmoud et al., 2019).

### 2.4 Immunoassays

Immunoassays for Aβ1‐40 (ThermoFisher, KHB3481), Aβ1‐42 (ThermoFisher KHB3441), aggregated Aβ (ThermoFisher, KHB3491) and sAβPP‐α (MyBioSource, MBS9358454) in CM was measured via ELISA according to manufacturer's instruction. Media samples were first concentrated using Amicon® ultra‐15 centrifuge filter units (3 kDa UFC9003, Millipore). IL‐6 and IL‐8 levels in conditioned media were quantified using Quantikine immunoassays (HS600C and D8000C, R&D systems). GFAP was measured in CM using Human GFAP DuoSet ELISA (R&Dsystems, DY2594‐05) as per manufacturer's instructions.

#### 2.4.1 Flow cytometry

To quantify intracellular accumulation of IL‐6 and IL‐8, astrocytes (1 × 10 (Magistretti & Allaman, 2015)) were incubated with protein transport inhibitor containing Brefeldin A (555 029 Golgiplug, BD Biosciences) and Monensin (554 724 Golgistop, BD Biosciences) following 48 h treatment with Aβ oligomers or 4 h with IL1β (10 ng/mL, 200‐01B Peprotech). Astrocytes were then dissociated and washed two times with D‐PBS before fixing and permeabilisation for 20 min (554 714 Cytofix/Cytopearm, BD Biosciences). Astrocytes were washed a further two times in wash buffer before incubation with IL‐6‐PE (12‐7069‐82, Invitrogen) and IL‐8 FITC (BH0814, BioLegend) conjugated antibodies for 30 min. Following a final three washes, astrocytes were analysed using a flow cytometer. Fluorescent compensation was applied using Ultracomp ebeads (01‐2222‐42, Invitrogen) and unstained astrocytes.

#### 2.4.2 ADAM10 activity

ADAM10 activity was measured via fluorometric FRET assay following manufacturer's instructions (AS‐72226, Sensolyte 520, Anaspec) and as previously described (Smith et al., 1985). ADAM10 activity was calculated using linear regression in relative fluorescence units (RFU) compared to 5‐FAM peptide activity.

#### 2.4.3 Oxidative stress measurements

Superoxide generation via MitoSOX™ Red mitochondrial superoxide indicator (M36008, Invitrogen) and lipid peroxidation via total F2 isoprostanes (8‐isoprostane ELISA kit, Cayman Chemical) were quantified as previously described (Elsworthy, Crowe, et al., 2022). Protein Carbonylation was assessed by the method of Carty et al. (2000). Briefly, cell lysates and standards (BSA) were added to carbonate buffer (sodium carbonate 50 mM, pH 9.2) and plated into 96 well plates (50 μL at 0.05 mg/mL) in triplicate. Proteins were allowed to bind for 1 h at 37°C before washing with TBS–Tween (0.5%). DNPH was added in HCl (1 mM) and allowed to react for 1 h at room temperature before washing with TBS–Tween (0.5%). Non‐specific binding sites were blocked overnight at 4°C with TBS‐Tween (1%). After washing, rabbit anti‐DNPH primary antibody (1:1000) was applied and incubated for 1 h at 37°C and, following washing with TBS‐Tween (1%), anti‐rabbit IgE conjugated to peroxidase (1:5000) was also incubated at 37°C for 1 h. The reaction was visualised by o‐phenylenediamine tablets with hydrogen peroxide (final conc. 7.8 mM) in citrate–phosphate buffer (10 mL) and stopped by addition of sulphuric acid (2 N). Absorbance was read at 490 nm (Fluostar Omega, BMG Labtech).

#### 2.4.4 Metabolites extraction and analysis

Metabolite extraction and profiling were performed as previously described (Surrati et al., 2016). Briefly, at day 50 in culture, cells (5 × 10^5^) were seeded in 6 well plates and maintained for 48 h before a full media exchange was completed. Following a further 48 h, 1 mL of the conditioned medium in each culture, was collected and centrifuged at 200 *g* for 5 min. 250 μL of the collected CM were transferred to a new microcentrifuge tube for extraction and protein precipitation. 750 μL of ice‐cold methanol was added and vortexed for 1 min and incubated at −20°C for 20 min to precipitate proteins. Samples were then vortexed again for 15 s and centrifuged at 16 000 *g*  for 10 mins at 4°C. The supernatant was transferred into precooled fresh tubes and samples were stored at −80°C until LC–MS analysis. Metabolite extraction from control, mutant astrocytes and Aβ‐treated control astrocytes were prepared and processed in parallel, as well as no cells controls (blanks). A pooled quality control (QC) sample was prepared by mixing equal volume from each sample (excluding blanks) to evaluate the robustness, performance and stability of the analytical system. Experimental and blank samples were randomised before injection. QC samples were included throughout the analysis to check the performance of the analytical system. Five mixed authentic standards solutions (total of 268 standards) were injected under the same condition for metabolite identification.

LC‐MS‐based metabolite profiling was performed on an Dionex Ultimate 3000 HPLC system coupled to a Q‐Exactive Plus hybrid quadrupole‐Orbitrap mass spectrometer (Thermo Fisher Scientific, UK) as previously described (Abdelrazig et al., 2020). Samples were separated on a ZIC‐pHILIC column (5 μm, 4.6′150 mm) from Merck Sequant (Watford, UK), with the mobile phases of 20 mM ammonium carbonate (A) and acetonitrile (B). Starting with 20%, mobile phase A increased linearly to 95% within 15 min before decreasing back to 20% in 2 min, where it was held for 7 min for equilibration. The injection volume was 10 μL and the flow rate was 300 μL/min. The chamber of autosampler was kept at 4°C and column temperature was maintained at 45°C. Mass spectrometer, fitted with HESI source, rapidly switched between positive (ESI+) and negative (ESI‐) mode with the spray voltage +4.5 and −3.5 kV, respectively. Other settings were optimised as follows: capillary temperature, 275°C; sheath gas flow rate, 40 arb; aux gas flow rate, 5 arb; sweep gas flow rate, 1 arb; S‐lens RF level, 55%. The samples were acquired using full MS scan method, ranging m/z 70 ~ 1050 at 70000 resolution. Top 10 data‐dependent MS/MS (ddMS/MS) was performed on QC samples at resolution of 17 500 and stepped normalised collision energy of 20, 30 and 40.

LC–MS data were analysed with Compound Discoverer 3.3 SP1with an untargeted metabolomic workflow (Thermo Fisher Scientific, UK) for peaks picking, peaks alignment, gap filling and metabolite identification. Compound annotation was made using exact mass (5 ppm error) using Human Metabolome Database (HMDB), Kyoto Encyclopedia of Genes and Genomes (KEGG), the accurate mass and retention time of authentic standards and *mz*Cloud fragmentation database.

According to the metabolomics standards initiative and scale (Sumner et al., 2007), the metabolites with high identification confidence (Level 1 and Level 2) were included and reported. In this study, Level 1 identification was that metabolites were matched with accurate masses, retention times and MS/MS fragmentation of authentic standards. Level 2 identification was detected peaks matched with accurate masses and MS/MS information of compounds in spectral library when lack of standards.

All identified compounds were subjected to pathways analysis using the online platform of MetaboAnalyst 5.0. Non‐human putative metabolites were excluded. The abundances of metabolites were log‐transformed and Pareto scaled before mapping the KEGG pathways and generating networks of interacting biological entities.

### 2.5 Statistical analysis

An a priori power analysis was conducted using G*Power version 3.1.9.6 (Faul et al., 2009) for sample size estimation, based on previous data (Elsworthy et al., 2021; Elsworthy, Crowe, et al., 2022). With a significance criterion of *α* = 0.05 and power = 0.80, the minimum sample size needed is *N* = 3 for measured outcomes. All quantitative data in the text and figures are presented as mean ± SD, unless otherwise stated. To generate a sigmoidal dose–response curve for Aβ treatment or standard curves from plate‐based assays either linear regression or four‐parameter logistic regression was used to plot known concentrations against optical absorbance at specified wavelengths. From this sample concentrations were calculated and normalised to total protein concentration in corresponding cell lysate. Significance was calculated using individual t‐tests for grouped data or using ordinary one‐way ANOVA with Bonferroni post hoc corrections and using linear regression models. The ROUT method (*Q* = 1%) was applied to identify outliers (Motulsky & Brown, 2006). All data were processed using GraphPad Prism (Version 9.3.1).

For metabolomics analysis, data were log‐transformed, and Student *t*‐test *p* value was adjusted with false discovery rate using Benjamini–Hochberg approach, compensating for the multiple testing problem. Adjusted *p* < 0.05 was regarded significant. Multivariate analysis, including principal component analysis (PCA) and orthogonal partial least squares discriminant analysis (OPLS‐DA) was performed on SIMCA‐P v16 (Umetrics, Sweden) to determine significant metabolites between each sample groups. After log transformation, the method of UV scaling and Pareto Scaling were used for PCA and OPLS‐DA respectively to make data more reasonable and comparable.

## 3 RESULTS

### 3.1 iPSC‐derived astrocytes display functional metabolic glycogen mobilisation in response to hypoglycaemia and adrenergic receptor activation treatment

**FIGURE 1:**
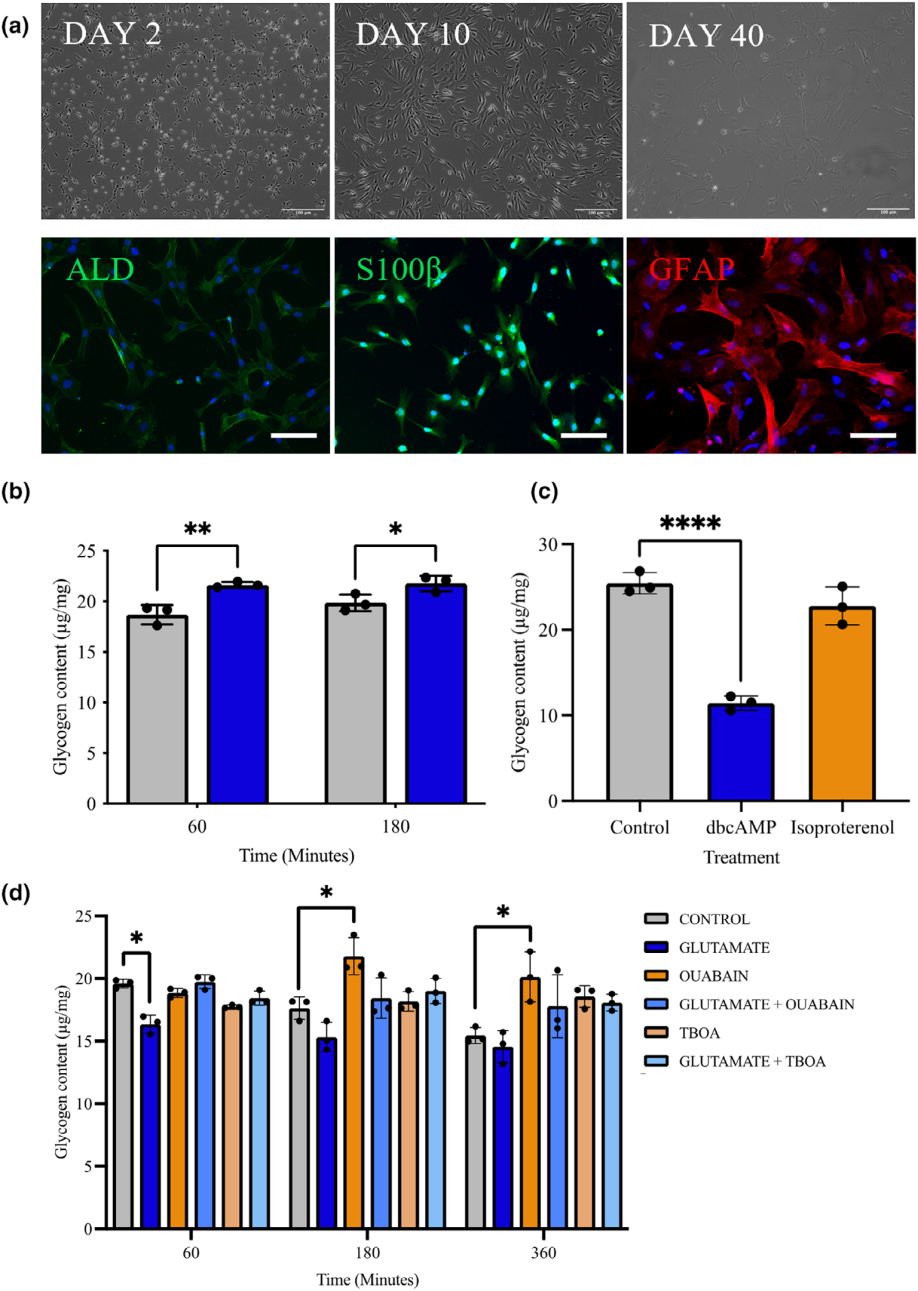
Characterisation of ‘healthy’ iPSC‐derived astrocytes. (a) Representative images showing differentiation and ICC staining of iPSC‐derived astrocytic cells at Day 45+. The astrocytes were differentiated from ‘healthy’ control NPCs for >40 days using astrocytes differentiation and maturation protocols. Top) Phase contrast images of control astrocytes on Days 2, 10 and 40 in culture post differentiation. Bottom) Cells were stained using immunofluorescent antibodies for astrocytic markers ALDH1L1 (left, green), S100β (middle, green) and GFAP (right, red), nuclei were counterstained in each image with DAPI (blue). Scale bars: 100 μM. (b) Cellular glycogen content of control astrocytes following exposure to hypoglycaemic conditions and treatment with DAB over 60 and 120 min. (c) Glycogen content of cells treated with dbcAMP an isoproterenol. (d) Glycogen contents of cells treated with Glutamate, oubain, glutamate and ouabain, TBOA and glutamate and TBOA for 60, 180 and 360 min. Results are expressed as ug/mg protein ± SD, *n* = 3 *p* < 0.05 (*), *p* < 0.01 (**), *p* < 0.001 (***). For DAB (b), a two‐way analysis of variance (ANOVA) was performed follow by Sidaks post‐test. Comparisons between treatments (c) were performed using analysis of variance (ANOVA) followed by Dunnet's post‐test. Each replicate or ‘*n*’ represents an independent culture preparation and is displayed as an individual data point.

To confirm differentiation of astrocytes from hNPCs, cellular changes in morphology was monitored via phase‐contrast imaging. Astrocytes were identified and stained using ICC for the astrocytic markers S100β, GFAP and ALDH1L1 at DAY 45+ (Figure 1a).

To determine whether functional glycogen stores were present in iPSC‐astrocytes, control cells were cultured under glucose‐starvation conditions to induce intracellular glycogen breakdown and with inhibitors of glycogen breakdown. A two‐way ANOVA was performed to analyse the effect of glucose starvation and DAB (glycogen phosphorylase and synthase inhibitor) on glycogen content of cells (*F*(1, 4) = 65.64, *p* = 0.001). Glycogen breakdown was significantly blunted with differences in cellular glycogen content from 19.59 ± 0.20 μg/mg in the control at 60 min (*p* = 0.007) to 21.64 ± 0.16 μg/mg in the presence of DAB and from 17.63 ± 0.52 μg/mg in the control at 180 min to 21.78 ± 0.44 μg/mg (*p* = 0.001; Figure 1b).

Next, to stimulate glycogen breakdown, control astrocytes were incubated with Isoproterenol (a β1 and β2 adrenergic agonist) or dbcAMP (an inducer of glycogen phosphorylase activity) for 180 min (to allow time for glycogen breakdown). Cellular glycogen content was assessed after 180 min via a one‐way ANOVA which revealed significant effects (*F*(2, 6) = 69.37, *p* = 0.0001). dbcAMP‐induced significant reduction in glycogen content from 25.44 ± 1.25 μg/mg, to 11.44 ± 1.17 μg/mg (*p* = 0.0001); however, there was no significant change in glycogen storage following treatment with isoproterenol (Figure 1c).

Given the key role astrocytes play in the homeostasis of the neurotransmitter glutamate levels and the energy cost associated with glutamate uptake, the effect of glutamate on glycogenolysis was assessed. Astrocytes were treated with glutamate (1 mM) with and without ouabain (100 μM, an inhibitor of Na^+^/K^+^ ATPase) and TBOA (100 μM, glutamate transporters inhibitor). Intracellular glycogen levels were measured at 60, 180 and 360 min corresponding to the timescale of glycogen breakdown to occur in astrocytes (Jakobsen et al., 2021) via a two‐way ANOVA (*F*(5, 12) = 11.57, *p* = 0.0003).

Across the treatments there was a significant effect of time with lowering glycogen levels in the control and glutamate‐treated astrocytes (*F*(1.5, 18.5) = 6.089, *p* = 0.014). Glutamate treatment induced significant reduction in glycogen levels in control astrocytes after 60 min (19.59 ± 0.24 μg/mg vs. 16.34 ± 0.51 μg/mg, *p* = 0.033). Treatment with ouabain or TBOA with and without glutamate blocked the effect of glutamate on glycogenolysis (*p* > 0.05; Figure 1d). The results are consistent with the physiological situation where glutamate uptake by EAATs is dependent on the electrochemical gradient maintained by the Na/K ATPase and additionally that astrocyte glycogen can be a source for ATP.

### 3.2 Amyloid‐beta oligomer treatment induces metabolic alterations and impaired glutamate uptake in iPSC‐derived astrocytes

Next, the effect of Aβ, on astrocyte glycolytic metabolism was observed. Here treatment with Aβ1‐42 oligomers, as a model for exposure to Aβ in AD, was investigated (Figure S2). First, the effects of Aβ1‐42 oligomers on cellular metabolic enzyme activity using MTT and viability of control astrocytes with AOPI probes were observed (Figure S2).

A dose‐dependent response (*F*(7, 32) = 8392, *p* = 0.0001) showed that 4 h treatment with Aβ1‐42 oligomers (0.078‐5 μM) caused a significant reduction in metabolic enzyme activity (78.77 ± 6.57%, *p* < 0.02) at 0.625 μM compared to control (Figure S2). This was with a maximum activity of 104.60%, minimum activity of 66.41% and EC50 of 0.571 μM. After 48 h treatment with Aβ1‐42 oligomers (0.078‐5 μM), there was a significant reduction (*F*(7, 32) = 19.7, *p* = 0.0001) in metabolic enzyme activity (79.33 ± 7.13%, *p* < 0.01) at 0.078 μM compared to control. This was with a maximum activity of 99.31%, minimum activity of 60.80% and EC50 of 0.099 μM. As MTT viability is reliant on mitochondrial function and enzymatic reduction of MTT, viability was also quantified, using AOPI dual fluorescence imaging which is an indication of cell membrane permeability (Figure S2). One‐way ANOVA analysis showed a significant treatment effect of Aβ1‐42 on viability (*F*(3, 8) = 19.67, *p* = 0.0005). Treatment of astrocytes with Aβ1‐42 oligomers significantly reduced cell viability at 0.2 μM (88.77 ± 1.468%, *p* = 0.014), 1 μM (88.77 ± 0.986% *p* = 0.014) and 2 μM (85.18 ± 0.745% *p* < 0.001) compared to control (92.20 ± 1.158%; Figure S2).

**FIGURE 2:**
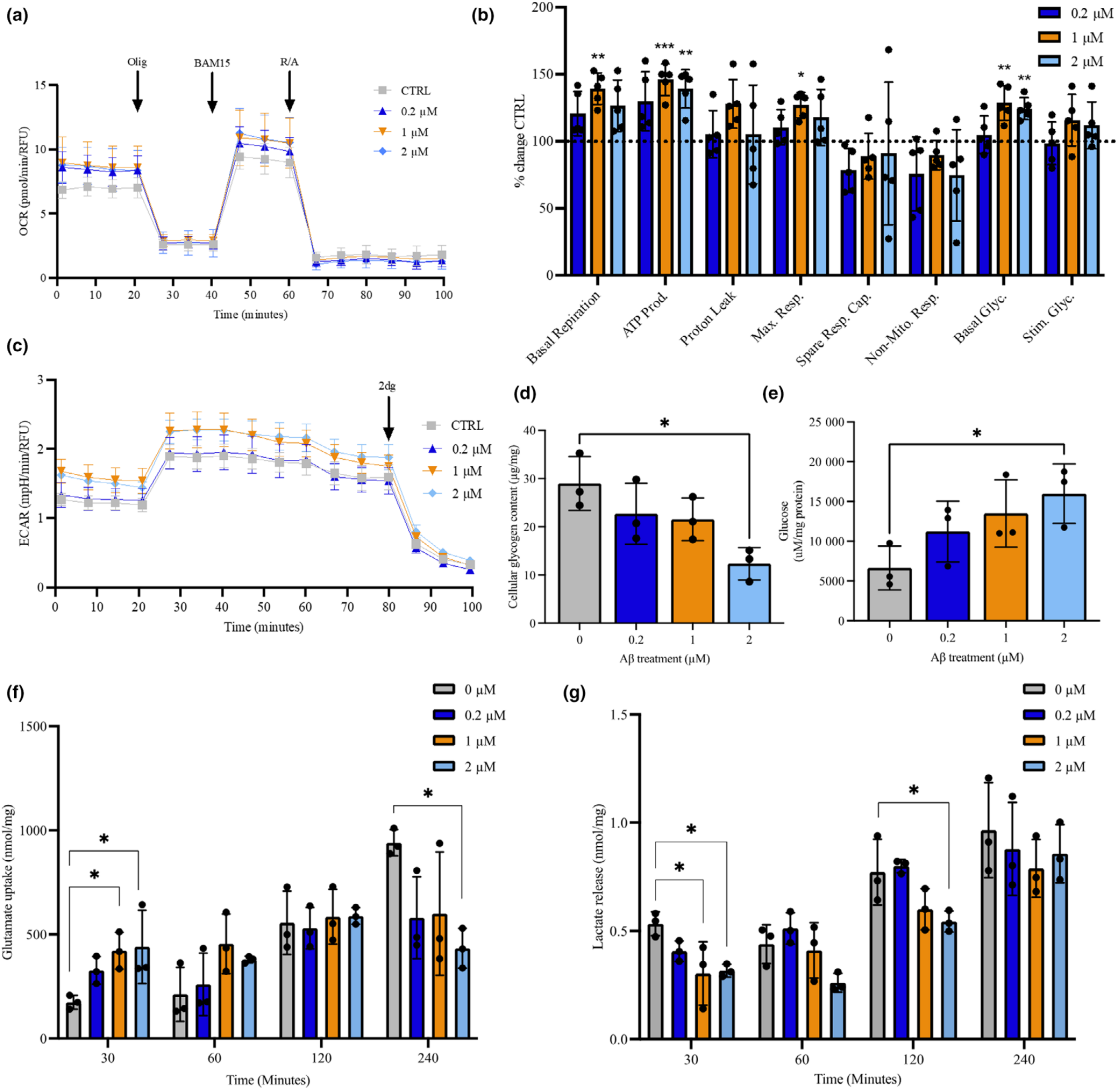
Amyloid β‐treated control astrocytes exhibit difference in their bioenergetic profiles and metabolite processing. Astrocytes (45 days old) were treated with 0.2, 1 and 2 μM Aβ oligomers. (a) Profiles of Seahorse XFp Mito Stress Test data for oxidative consumption rates (OCR, pmol/min). (b) Percentage change of treated cells over control (untreated) cells. (c) Extracellular acidification rate (ECAR, mpH/min) measured after treatment with 0.2, 1, 2 μM Aβ oligomers. (d) Cellular glycogen content of cells (μg/mg cellular protein). (e) Glucose levels remaining in the media μM/mg cellular protein, (f) Glutamate uptake (nmol/mg cellular protein), (g) Lactate release (nmol/mg cellular protein) after treatment with 0.2, 1, 2 μM Aβ oligomers. Results are expressed as ± SD, *n* = 3 *p* < 0.05 (*), *p* < 0.01 (**), *p* < 0.001 (***). Comparisons between treatments were performed using ANOVA followed by Dunnet's post‐test. Each replicate or ‘*n*’ represents an independent culture preparation and is displayed as an individual data point.

To understand whether cellular bioenergetic profiles were altered by Aβ1‐42 oligomers, metabolic activity was analysed in the astrocytes using Seahorse XF analyser to gain real‐time data for both oxygen consumption rate (OCR) and extracellular acidification rate (ECAR). Basal OCR (*F*(3, 16 = 6.059, *p* = 0.006)) was significantly increased following treatment with either 1 μM (9.691 ± 0.824 pmol/min, *p* = 0.001) or 2 μM (8.810 ± 1.33 pmol/min, *p* = 0.03) Aβ1‐42 compared to control (8.404 ± 1.145 pmol/min). Maximal respiratory capacity (*F*(3, 16) = 3.109, *p* = 0.0005) was significantly increased only at treatment with 1 μM Aβ1‐42 (11.68 ± 0.865 pmol/min, *p* = 0.025) as compared to vehicle‐treated (9.203 ± 1.102 pmol/min). ATP‐coupled respiration (*F*(3, 16) = 8.942, *p* = 0.001) was significantly increased at 0.2 μM (5.691 ± 0.960 pmol/min, *p* = 0.018), 1 μM (6.398 ± 0.513 pmol/min, *p* < 0.001) and 2 μM (6.101 ± 0.626 pmol/min, *p* = 0.002) Aβ1‐42 treatments compared to control (4.386 ± 0.429 pmol/min). There was no significant difference in proton leak, spare respiratory capacity or non‐mitochondrial oxygen consumption (Figure 2a,b).

For ECAR measurements, basal glycolysis (*F*(3, 16) = 8.082, *p* = 0.0017) was significantly elevated following treatment with both 1 μM (1.575 ± 0.584 mpH/min, *p* = 0.002) and 2 μM (1.524 ± 0.100 mpH/min, *p* = 0.008) Aβ1‐42 compared to control (1.225 ± 0.098 mpH/min). There was no significant difference in glycolytic rates between conditions following oligomycin injection. Glycolysis‐derived ATP levels (*F*(3,16) = 5.578, *p* = 0.0082) were significantly elevated following 1 μM (2.243 ± 0.223 pmol/min, *p* = 0.017) and 2 μM (2.231 ± 0.149 pmol/min, *p* = 0.002) Aβ1‐42 treatments compared to control (1.860 ± 0.134 pmol/min, *p* = 0.021; Figure 2c).

Based on our previous functional data, astrocytic glycogen levels were determined in cell lysates after 48‐h treatment with Aβ1‐42. There was a significant reduction in glycogen content (*F*(3, 8) = 5.511, *p* = 0.0239) following exposure with 2 μM Aβ1‐42 oligomers (12.36 ± 1.94 μg/mg, *p <* 0.01) compared to control (28.97 ± 3.23 μg/mg). No significant effects were observed following treatment with either 0.2 μM or 1 μM Aβ1‐42 (Figure 2d). To determine the effect of Aβ1‐42 oligomers on glucose uptake in astrocytes, the amount of glucose remaining in the media was monitored. Following treatment with Aβ1‐42 there was a significant increase (*F*(3,8) = 3.503, *p* = 0.0694) in glucose remaining in culture media after 48 h following 2 μM treatment (15 990 ± 3731 μM/mg, *p <* 0.035) compared to control (6644 ± 2756 μM/mg; Figure 2e). No significant difference in glucose levels in the media (*p* > 0.05) were detected at any other time point or concentration of Aβ1‐42 treatment.

Our data have demonstrated that astrocytes increase glycolysis and glycogenolysis after stimulation with glutamate in order to meet increased energetic demands. Given that utilisation of alternative energy sources may become essential in AD wherein levels of lactate become elevated in MCI and CSF of AD patients and glutamate levels may become perturbed, we next investigated the impact of Aβ (Hascup et al., 2022). To understand whether the effect of Aβ1‐42 treatments was associated with alterations in glutamate uptake and lactate release, astrocytes were treated with 200 mM glutamate and sampled at 30, 60, 120 min and 4 h. Glutamate and lactate levels in conditioned media were also collected after 48 h (Figure 2f). After 30 min, glutamate uptake was significantly elevated (*F*(3, 8) = 4.299, *p* = 0.0440) following 48‐h treatment with 1 μM (421.7 ± 88.41 μmol/mg, *p* = 0.004) and 2 μM (440.3 ± 175.8 μmol/mg, *p* = 0.031) Aβ1‐42 compared to control (164.1 ± 37.64 μmol/mg). There was no significant difference between glutamate uptake at both 60 and 120 min. Interestingly, by 4 h glutamate uptake was reduced following Aβ1‐42 treatments and was significantly lower than control (940.7 ± 63.25 μmol/mg) following 2 μM treatment (433.2 ± 95.57 μmol/mg, *p* = 0.026). Glutamate levels in CM were not significantly altered after 48 h (Figure 2f). Lactate release was significantly lower after 30‐min treatment with 200 mM glutamate (*F*(3, 8) = 4.889, *p* = 0.0323) after both 1 μM (0.304 ± 0.146 nmol/mg, *p* = 0.024) and 2 μM (0.317 ± 0.029 nmol/mg, *p* = 0.031) Aβ1‐42 treatments compared to control (0.533 ± 0.055 nmol/mg). After 60 and 120 min, lactate release remained lowered, however this was only significant at 120 min following 2 μM Aβ1‐42 treatment (0.544 ± 0.049 nmol/mg, *p* = 0.044) compared to control (0.772 ± 0.152 nmol/mg). There was no difference in lactate release after 4 or 48 h (Figure 2g).

### 3.3 iPSC‐derived astrocytes exposed to amyloid oligomers display features of reactive gliosis

**FIGURE 3:**
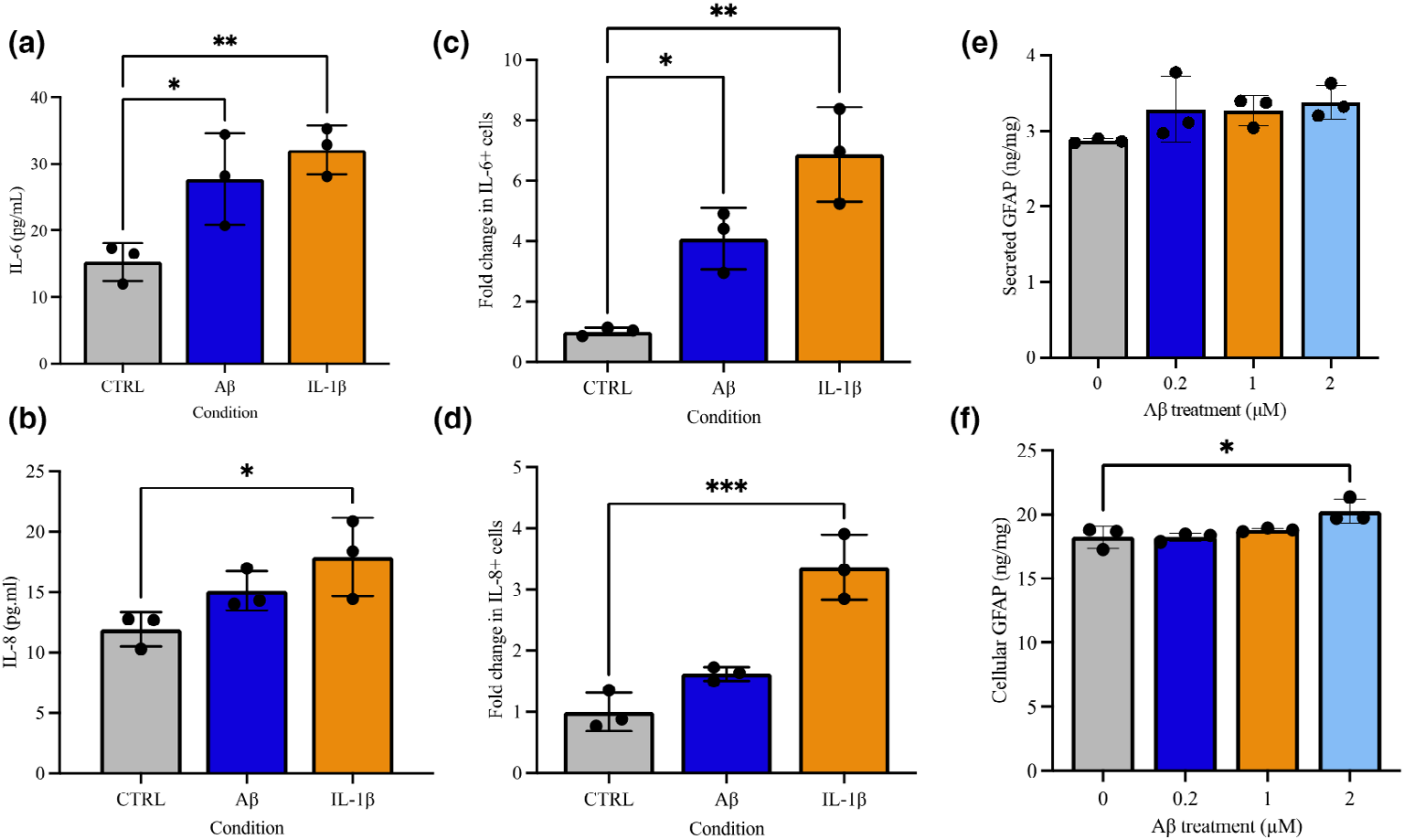
Astrocytes display markers of gliosis following exposure to Aβ oligomers. Control astrocytes were treated with Aβ oligomers for 48 h. Astrocytes (45 days old) were treated with 0.2, 1, 2 μM Aβ oligomers. Cytokine or GFAP levels in media were measure using ELISA or by investigating intracellular accumulation using flow cytometry or ELISA. (a) IL‐6 levels (pg/mL) in the media were measured using ELISA or (c) Flow cytometry following treatment of astrocytes with Aβ oligomers. (b) IL‐8 levels (pg/mL) in the media were measured using ELISA or (d) Flow cytometry (fold change) following treatment of astrocytes with Aβ oligomers. (e) Secreted levels of GFAP in the media or (f) GFAP in cell lysates were measured using ELISA (ng/mL). Results are expressed as ± SD, *n* = 3 *p* < 0.05 (*), *p* < 0.01 (**), *p* < 0.001 (***). Comparisons between treatments were performed using ANOVA followed by Dunnet's post‐test. Each replicate or ‘*n*’ represents an independent culture preparation and is displayed as an individual data point.

As changes in metabolism are well‐known to drive changes in astrocyte phenotype from neuroprotective to neurotoxic (Jakobsen et al., 2021), we next investigated whether Aβ1‐42 treatment‐induced gliosis in astrocytes. One key feature of reactive gliosis is an increase in cell size (Hascup et al., 2022). Following treatment with Aβ1‐42 a detectable increase in cell diameter at 0.2 μM (10.57 ± 0.351 microns, *p* = 0.018), 1 μM (10.67 ± 0.635 microns, *p* = 0.012) and 2 μM (10.90 ± 0.265 microns, *p* = 0.004) compared to control (9.433 ± 0.058; see Figure S2). The addition of IL‐1β to astrocytes for 48 h resulted in the significantly increased secretion of IL‐6 ((*F*(2, 6) = 9.991, *p* = 0.0123) 32.09 ± 3.642 pg/mg, *p* = 0.04) and IL‐8 ((*F*(3, 6) = 5.342, *p* = 0.0465) 17.89 ± 3.240 pg/mg, *p* = 0.03) as compared to control (15.27 ± 2.877 pg/mg and 11.90 ± 1.410 pg/mg, respectively). With 48 h treatment of 2 μM Aβ1‐42, astrocytes showed a significant increase in IL‐6 (27.76 ± 6.871 pg/mg, *p* = 0.02) but not for IL‐8 (15.10 ± 1.636 pg/mg, *p* > 0.05; Figure 3a,b). Further evidence of an altered immune state was gathered by quantifying the accumulation of IL‐6 and IL‐8, following treatment with IL1‐β and addition of protein transport inhibitors, as compared with vehicle‐treated condition. A one‐way ANOVA revealed significant effects for both IL‐6 (*F*(2, 6) = 21.96, *p* = 0.0017) and IL‐8 (*F*(2, 6) = 34.48, *p* = 0.0005). IL1‐β treatment significantly increased both intracellular IL‐6 (6.865 ± 1.569 FC, *p* = 0.001) and IL‐8 (3.360 ± 0.530 FC *p* < 0.001) in comparison with control (values expressed fold change to control, Figure 3c,d). In response to Aβ1‐42 treatment, IL‐6 (4.080 ± 1.019 FC, *p* = 0.02) was significantly elevated, but this was not seen for IL‐8 (1.618 ± 0.113 FC, *p* > 0.05) compared to control matching cytokine secretion data.

In addition to cytokine markers, GFAP levels was also quantified in both media and cell lysates using an ELISA and by quantifying ICC protein expression, this time for a range of Aβ concentrations. GFAP is increasingly recognised as a marker of neurodegenerative disorders as well as traumatic brain injury and is upregulated during gliosis in response to chemical, biological insults as well as brain trauma (Kwart et al., 2019). A one‐way ANOVA (*F*(3, 8) = 6.201, *p* = 0.0175) showed there was no significant difference in secreted GFAP in CM following 48 h of Aβ treatment; however, 2 μM treatment with Aβ significantly elevated intracellular GFAP protein levels (20.26 ± 0.952 ng/mg) compared to control (18.25 ± 0.851 ng/mg). GFAP protein expression was significantly higher following 2 μM treatment with Aβ compared to control (Figure S3). Quantification of mitochondrial superoxide generation relative to control (fold change, FC) using a one‐way ANOVA (*F*(3, 12) = 4.325, *p* = 0.0276) showed a dose depended lowering following treatment with 2 μM Aβ1‐42 (0.837 ± 0.054 FC, *p* = 0.015; Figure 3e,f).

### 3.4 Altered AβPP processing in astrocytes carrying PSEN1 mutations

**FIGURE 4:**
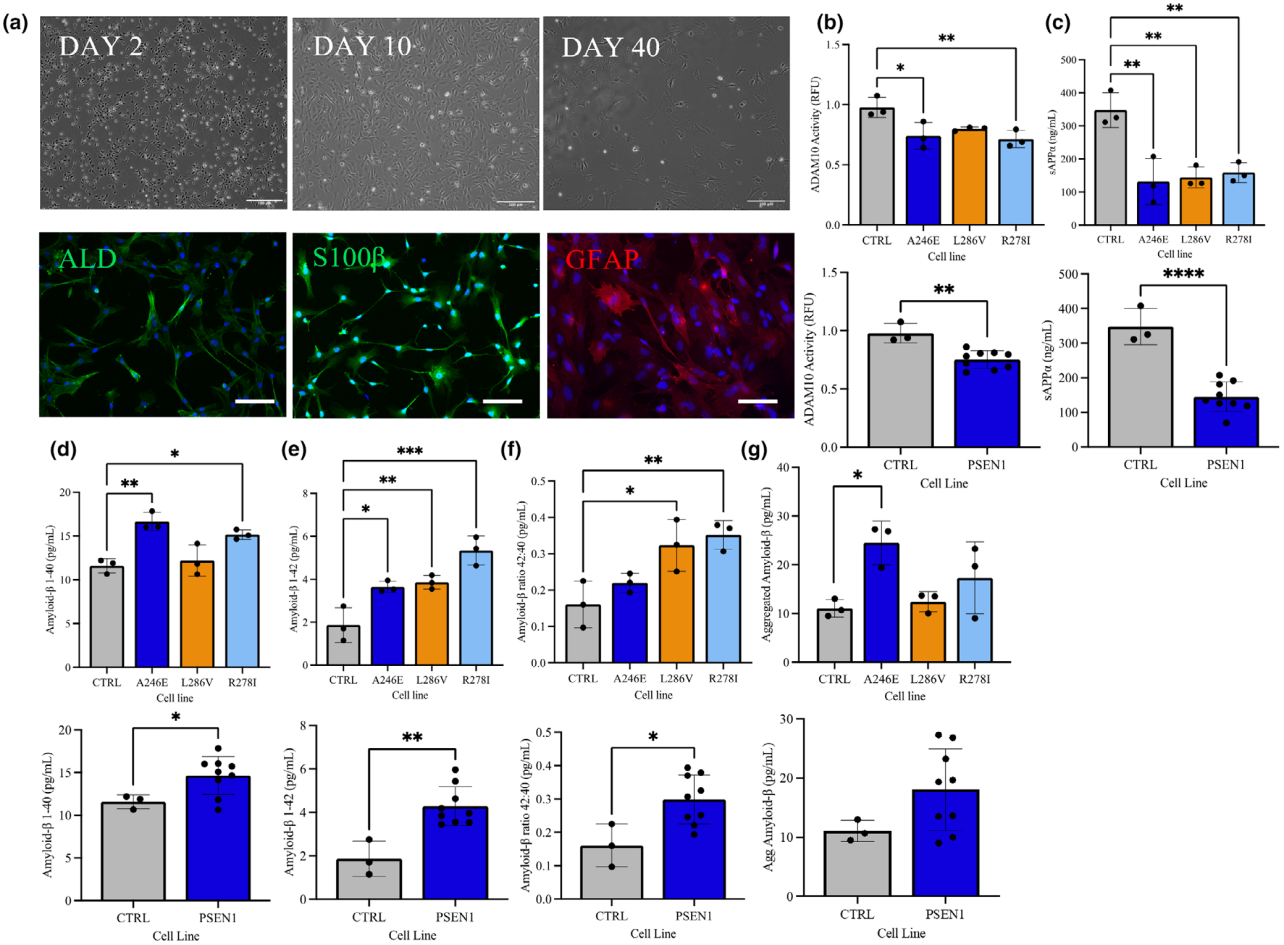
Astrocytes process APP differentially in ‘healthy’ control versus fAD patient‐derived cells. Control and fAD (PSEN1 mutation)‐derived astrocytes (Day 45) were characterised using immunofluorescence and APP processing was assessed using ELISA and ADAM 10 activity. (a) Representative images of fAD (PSEN1 mutation) derived astrocytes. Cells were stained using immunofluorescent antibodies for astrocytic markers ALDH1L1 (green), S100β (Green) and GFAP (red). nuclei were counterstained with DAPI (blue). Scale bars: 100 μM. (b) Characterisation of ADAM10 enzymatic activity (RFU) and (c) soluble APPα (ng/mL) in control and PSEN1 (A246E, L268V and R278I). Pooled PSEN1 samples compared to control are displayed. (d) Aβ 1–40 (pg/mL), (e) Aβ 1–42 (pg/mL), (f) Aβ42/40 ratio, (g) aggregated Aβ (pg/mL) were measure in control and fAD patient‐derived astrocytes after 48 h. Pooled PSEN1 samples compared to control are displayed. Results are expressed as ± SD, *n* = 3 *p* < 0.05 (*), *p* < 0.01 (**), *p* < 0.001 (***). For direct comparison between control and PSEN1, unpaired *t*‐tests were performed. Comparisons between individual PSEN1 lines were performed using ANOVA followed by Dunnet's post‐test. Each replicate or ‘*n*’ represents an independent culture preparation and is displayed as an individual data point.

While treatment with Aβ provides some insight into the impact of acute amyloid exposure, fAD iPSC‐derived astrocytes may provide a more physiological assessment of chronic APP misprocessing and exposure to Aβ. Although PSEN1 mutations are typically associated with a shift towards longer Aβ isoform accumulation, there is evidence of differences between mutation sites (Kwart et al., 2019). Thus, ‘pooled’ PSEN1 lines and individual lines were compared to controls. To quantify AβPP processing in fAD iPSC derived astrocytes carrying PSEN1 mutations compared to ‘healthy’ control, markers associated with amyloidogenic and non‐amyloidogenic processing such as Aβ1‐40 and Aβ1‐42 levels were measured. As expected, Aβ1‐40 was significantly elevated in pooled PSEN1 astrocytes compared to control astrocytes (14.67 ± 2.230 vs.11.58 ± 0.806 pg/mg, *p* = 0.055) as quantified by ELISA. A one‐way ANOVA showed significant effects between cell lines (*F*(3, 8) = 13.37, *p* = 0.0018). Analysis of iPSC derived astrocytes carrying a R278I mutation (15.16 ± 0.543 pg/mg, *p* = 0.012) and A246E (16.64 ± 1.042 pg/mg, *p* = 0.002) mutations demonstrated significantly elevated Aβ1‐40 compared to control astrocytes; however, there was no detectable difference in the L286V mutation (12.20 ± 1.778 pg/mg, *p* > 0.05; Figure 4d). Aβ1‐42 was significantly elevated in PSEN1 astrocytes compared to control (4.283 ± 0.892 vs. 1.868 ± 0.810 pg/mg, *p* = 0.002). This was observed in all PSEN1 mutations (*F*(3, 8) = 18.97, *p* = 0.0005), R278I (5.342 ± 0.672 pg/mg, *p* = 0.002), L286V (3.861 ± 0.319 pg/mg, *p* = 0.007) and A246E (3.645 ± 0.266 pg/mg, *p* = 0.012) compared to control (Figure 4e). In AD, the ratio Aβ1‐42:40 is elevated, we next tested whether this was also seen in PSEN1 astrocytes. Taking the ratio of Aβ1‐42:40, there was an increase in PSEN1 astrocytes compared to control (0.299 ± 0.074 vs. 0.1607 ± 0.064 pg/mg, *p* = 0.017). Interestingly, this ratio was not elevated in the A246E mutation, however, aggregated‐Aβ was significantly increased at baseline in A246E (24.51 ± 4.461 ng/mL, *p* = 0.02) astrocytes only, compared to control (11.07 ± 1.781 ng/mg; Figure 4f,g). Together, this suggests that fAD‐associated mutations to PSEN1 in astrocytes affect APP processing and therefore will enable us to determine the impact of altered amyloid production of metabolic and disease associated phenotypes.

To understand how non‐amyloidogenic AβPP processing is affected in astrocytes carrying PSEN1 mutations, ADAM10 activity and sAβPPα were quantified. ADAM10 activity was significantly lower in PSEN1 astrocytes compared to control (0.978 ± 0.084 vs. 0.751 ± 0.076 RFU *p* = 0.001). A one‐way ANOVA showed this (*F* (3, 8) = 6.850, *p* = 0.0134) was significantly lower in R278I (0.715 ± 0.071 RFU, *p* = 0.009) and A246E (0.741 ± 0.112 RFU, *p* = 0.02) mutations compared to control. Additionally, sAβPPα (*F*(3, 8) = 13.17, *p* = 0.0018) was significantly lower in R278I (158.7 ± 29.64 pg/mg, *p* = 0.004), L286V (144.5 ± 31.63 pg/mg, *p* = 0.002) and A246E (131.7 ± 70.00 pg/mg, *p* = 0.002) compared to control (347.9 ± 52.15 pg/mg; Figure 4a,b).

### 3.5 PSEN1 carrying astrocytes show features of altered metabolism and glutamate uptake

**FIGURE 5:**
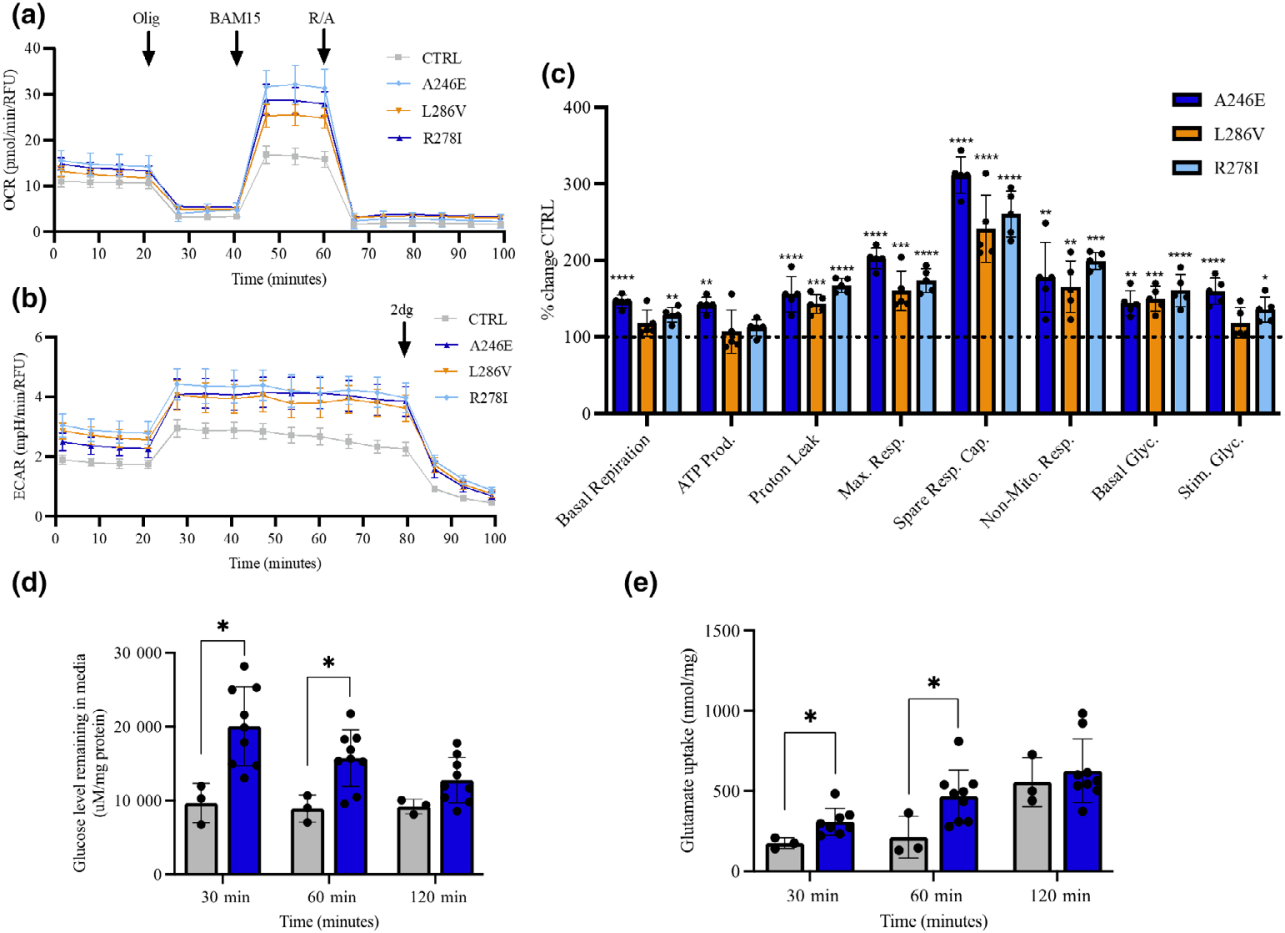
fAD‐derived astrocytes exhibit differences in their bioenergetic profiles and metabolite processing compared to ‘healthy’ control cells. (a) Profiles of Seahorse XFp Mito Stress Test data for oxidative consumption rates (OCR, pmol/min). (b) Extracellular acidification rate (ECAR, mpH/min). (c) Percentage change of fAD cells over control. (d) Glucose levels remaining in the media (μg/mg cellular protein), (e) Glutamate uptake (nmol/mg cellular protein) following addition of glutamate (200 μM) over 30, 60 and 120 min. Results are expressed as ± SD, *n* = 3 *p* < 0.05 (*), *p* < 0.01 (**), *p* < 0.001 (***). Comparisons between treatments were performed using ANOVA followed by Dunnet's post‐test. Each replicate or ‘*n*’ represents an independent culture preparation and is displayed as an individual data point.

As treatment with Aβ1‐42 oligomers resulted in a significant shift in metabolic profile, we investigated the profiles of PSEN1 carrying astrocytes compared to healthy controls, OCR and ECAR were quantified using Seahorse assay. Basal OCR was elevated in PSEN1 astrocytes compared to control (14.11 ± 1.789 vs. 10.79 ± 1.181 pmol/min, *p* = 0.001); however, when looking at individual mutations (*F*(3, 16) = 13.08, *p* = 0.0001), this was only altered in R278I (13.88 ± 1.031 pmol/min, *p* = 0.004) and A246E (15.75 ± 0.880 pmol/min, *p* < 0.001) mutations but not in L286V. After correcting for basal OCR, maximal respiratory capacity was significantly elevated in all PSEN1 mutations compared to control (15.12 ± 2.428 vs. 5.580 ± 0.722 pmol/min, *p* < 0.001). Proton leak (5.065 ± 0.580 vs. 3.260 ± 0.372 pmol/min, *p* = 0.001) and non‐mitochondrial oxygen consumption (3.114 ± 0.588 vs. 1.721 ± 0.317 pmol/min, *p* < 0.001) was also significantly elevated in PSEN1 mutations compared to control, whereas ATP linked respiration was only elevated in A246E astrocytes. Not only was OCR elevated, but there was also an increase in ECAR at baseline in PSEN1 mutants compared to controls indicating a greater overall cell energy demand (*t*(18) = 6.068, 2.717 ± 0.325 vs. 1.794 ± 0.146 mpH/min, *p* < 0.001). Glycolytic rate, corrected for or basal rates, was also elevated in PSEN1 astrocytes following the addition of oligomycin to astrocytes (*t*(18) = 3.317, 1.519 ± 0.266 vs. 1.103 ± 0.132 mpH/min, *p* = 0.001; Figure 5a,c).

To understand whether the PSEN1 mutations were associated with functional metabolic processes such as glutamate uptake and lactate release, astrocytes were incubated with media containing 200 mM glutamate and sampled at 30, 60 and 120 min Following glutamate treatment PSEN1 astrocytes displayed an elevated uptake of glutamate at 30 min (*t*(9) = 2.606, 307.7 ± 83.91 vs. 174.2 ± 33.32 μmol/mg, *p* = 0.028) and 60 min (*t*(10) = 2.407, 466.3 ± 164.5 vs. 212.5 ± 130.0 μmol/mg, *p* = 0.038) post treatment; however, this was not significantly different to control at 120 min. Glutamate remaining in cell culture media after 48 h was significantly elevated in PSEN1 astrocytes compared to control (*t*(10) = 3.487, 2723 ± 345.5 vs. 1968 ± 222.6 μmol/mg, *p* = 0.006). Glutamate uptake can be a highly energy consuming process due to the additional ATP demands of exchanging Na^+^ and K^+^ ions. Thus, glucose uptake from cell culture media and lactate release were measured. Interestingly, despite elevated glutamate uptake there was no significant change in lactate release for any time points, however, glucose remaining in cell culture media was elevated at 30 min (*t*(10) = 3.164, 20 048 ± 5349 vs. 9648 ± 2661 μmol/mg, *p* = 0.010) and 60 min (*t*(10) = 2.906, 15 729 ± 3828 vs. 8912 ± 1814 μmol/mg, *p* = 0.016) post glutamate treatment. There was also significantly more glucose remaining in cell culture media at 48 h in PSEN1 astrocytes compared to controls (*t*(10) = 3.222, 11 388 ± 2049 vs. 6644 ± 2756 μmol/mg, *p* = 0.009; Figure 5d,e).

### 3.6 PSEN1 carrying astrocytes display features of reactive gliosis and oxidative stress

**FIGURE 6:**
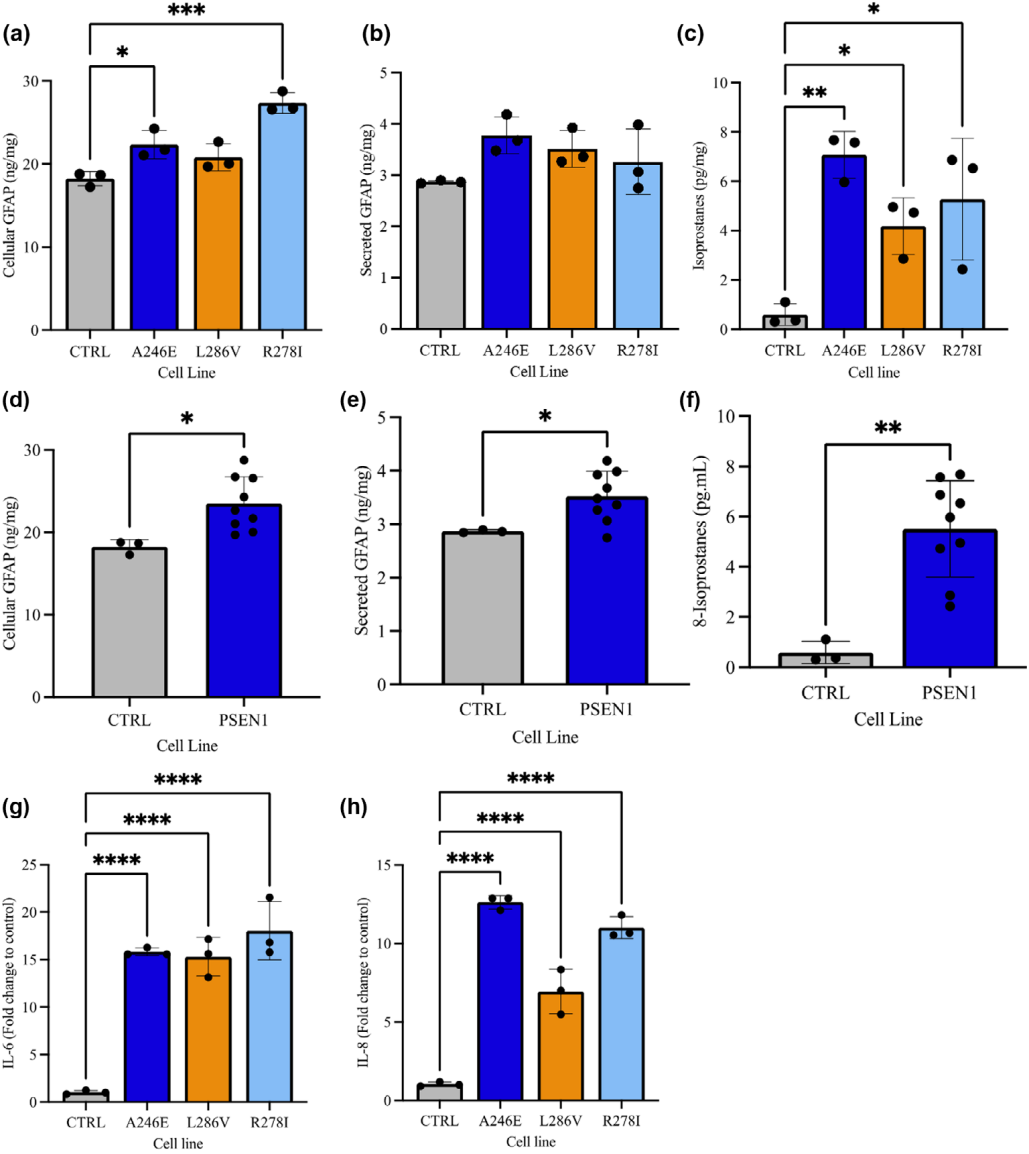
fAD astrocytes display markers of gliosis compared to ‘healthy’ control astrocytes. Cytokine, GFAP, 8‐isoprostane levels in media or cellular lysates were measured in control and fAD astrocytes (45 days old). (a) Levels of GFAP were measure in the cell lysates or (b) cell culture media using ELISA (ng/mL). (c) Isoprostane levels were also measured in cellular lysates (pg/mg). Pooled control and fAD cell samples were compared in (d) cellular GFAP and (e) secreted GFAP, (f) isoprostanes. Fold change in the accumulation of (g) IL‐6 and (h) IL8 following protein transport inhibition measured using flow cytometry. Results are expressed as ± SD, *n* = 3 *p* < 0.05 (*), *p* < 0.01 (**), *p* < 0.001 (***). For direct comparison between control and PSEN1, unpaired t‐tests were performed. Comparisons between individual PSEN1 lines were performed using ANOVA followed by Dunnet's post‐test. Each replicate or ‘*n*’ represents an independent culture preparation and is displayed as an individual data point.

Numerous studies have described reactive astrocytes in postmortem brain of patients with AD and pre‐symptomatically in mouse models of AD, suggesting that astrocytes are reactive in AD. As with measurements in Aβ1‐42‐treated astrocytes, markers associated with a reactive astrocytic state were quantified in PSEN1 mutation cells. A one‐way ANOVA showed (*F*(3, 8) = 1.444, *p* = 0.0002) PSEN1 astrocytes demonstrate a detectable increase in cell diameter for A246E (11.43 ± 0.12 microns, *p* = 0.001) and R287I (11.47 ± 0.23 microns, *p* = 0.0002) compared to control (9.47 ± 0.05 microns; see Figure S1). Measurements of GFAP secreted into cell culture media was significantly elevated in PSEN1 astrocytes (*t*(10) = 2.342, 3.523 ± 0.468 vs. 2.870 ± 0.028 ng.mg, *p* = 0.041), this was also seen in cell lysates compared to controls (*t*(10) = 2.688, 23.50 ± 3.249 vs. 18.25 ± 0.851 ng/mg, *p* = 0.023). Mitochondrial superoxide generation, measured via Mitosox Red was not significantly different between PSEN1 and control when grouped, although, one of the mutations, A246E, was significantly elevated compared to control (309 400 ± 23 669 vs. 223 455 ± 17 610 RFU, *p* = 0.0009) in specific PSEN1 mutation analysis. However, lipid oxidation measured as total F2 8‐isoprostane was significantly elevated in all PSEN1 mutant lines as compared to control (*t*(10) = 4.271, 5.510 ± 1.919 vs. 0.5907 ± 0.4420 pg/mg, *p* = 0.0016; Figure 6c,f). Flow cytometric analysis of cytokine accumulation under resting conditions were quantified following protein trafficking inhibition. Both IL‐6 (*t* (10) = 11.48, 16.29 ± 2.243 vs. 1.028 ± 0.120 FC, *p* < 0.001) and IL‐8 (*t* (10) = 5.760, 10.19 ± 2.663 vs. 1.036 ± 0.140 FC, *p* = 0.002) were significantly higher in PSEN1 astrocytes than controls, respectively (Figure 6d,e).

### 3.7 Astrocytes carrying fAD‐associated mutations present an altered metabolome

**FIGURE 7:**
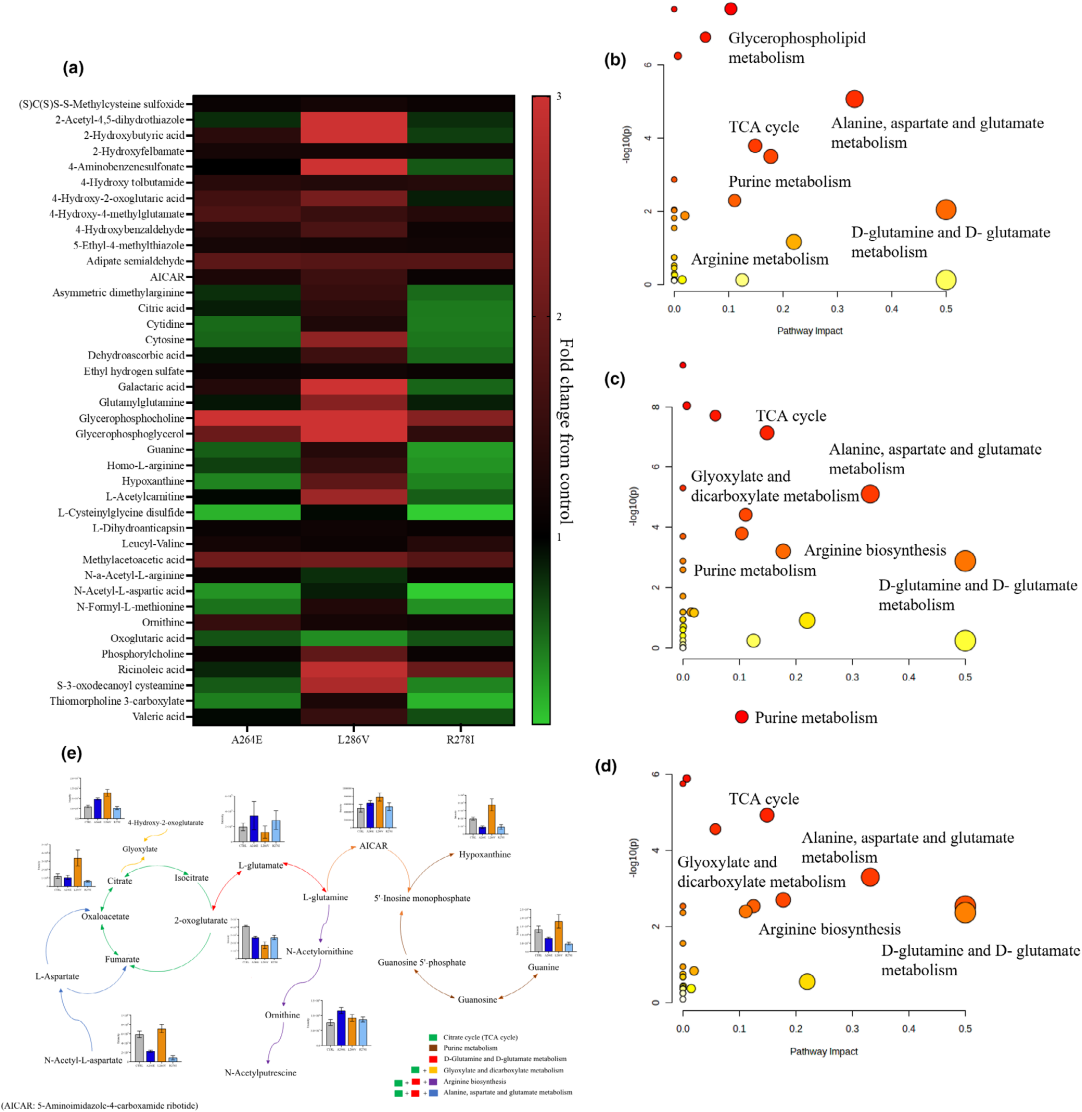
fAD‐derived astrocytes display significantly altered metabolic profiles compared to controls. (a) Heatmap displaying differential compounds identified using metabolomic analysis. Summary of pathway analysis for the comparison of control and b) A246E, (c) L286V and (d) R278 fAD patient‐derived astrocytes. The pathway analysis results of PSEN1 astrocytes compared with control. The colour graduated from white to and red indicates the degree of significance, the size of bubble represents the number of metabolites hit in the pathway. (e) Bar charts indicating intensity changes in key metabolites represented within metabolic pathway that were detected in astrocytes carrying PSEN1 mutations. Data shown are expressed as ± SD, *n* = 6. Each replicate or ‘*n*’ represents an independent culture preparation.

As metabolites including glucose, glutamate and lactate demonstrated altered release and uptake, metabolite profiling of astrocyte CM from cells carrying fAD mutation and controls was performed by the application of high‐resolution mass spectrometry. Based on the results of univariate analysis, metabolites with adjusted *p* < 0.05 and fold change >2 were selected as significant. The heatmap plots demonstrate a significantly altered abundance in metabolites in PSEN1 astrocytes compared to control, with L286V astrocytes displaying an altered metabolism to both A246E and R278I astrocytes (Figure 7a). For multivariate analysis, the PCA models were built to evaluate the similarities among the variables and the robustness of analytical system. The pooled QC samples were clustered tightly towards the centre of the scores plot, indicating that the satisfactory stability of instrument was achieved. Clear clustering and separation for different mutant groups and control groups were observed, suggesting that astrocytes carrying‐fAD mutations present a significantly different metabolic profiles as compared to controls (Figure S3a–d).

The OPLS‐DA models were next constructed to identify the differential compounds between the PSEN1 and control groups. On the OPLS‐DA scores plot, mutation and control groups were clearly clustered and separated, indicating the significant distinction of metabolic profile between each PSEN1 type and control astrocytes. Combined with cross‐validation results and permutation results, the models constructed for this study were less likely to overfit our dataset, with good fitness and good predictive ability (R^2^Y > 0.9 and Q^2^ > 0.9). The lists of variables importance in projection (VIP) were then generated for each comparison. VIP value generally represents the importance of variables in the OPLS‐DA model and has been used to extract the metabolites related to separation (Yin et al., 2009; Figure S4a–d). Finally, in this study, the metabolites with VIP >1 and adjusted *p* < 0.05 were considered as key compounds. As compared to control astrocytes, 8, 8 and 10 differential metabolites (Level 1 and Level 2) were significantly dysregulated in astrocytes carrying A246E, L286V and R278I, respectively.

To identify which metabolic pathways are dysregulated in AD astrocytes, pathway analysis was performed using the all the identified metabolites. Purine metabolism, alanine, aspartate and glutamate metabolism and citrate (TCA cycle) metabolism were the three most dysregulated pathways for astrocytes carrying A246E and R278I mutations. The metabolites mapped onto these pathways were significantly less abundant in astrocytes carrying A246E and R278I mutations than in control astrocytes. For astrocytes carrying the L286V mutation, different from A246E and R278I mutations, the metabolites involved purine metabolism, alanine, aspartate and glutamate metabolism, and TCA cycle were more abundant in L286V mutated astrocytes compared with control astrocytes. Due to a higher impact score on the pathway analysis, the D‐glutamine and D‐glutamate metabolism was selected to compare L286V mutations with control, with metabolites in this pathway (including L‐glutamate and 2‐oxoglutarate) significantly reduced as compared to control astrocytes. Together, the data presented demonstrate that astrocytes carrying AD mutations present a dysregulated metabolism and that different AD mutations can have different effects on astrocyte metabolism (Figure 7b,e).

## 4 DISCUSSION

Astrocytes are key mediators in cerebral metabolism and may even carry a similar metabolic cost to neurons which are typically thought of as the most energetically demanding cells in the brain (Barros, 2022; Beard et al., 2022). Disruption of astrocytic function, either caused by, or resulting in, a reduction in glucose uptake, may result in a decline in homeostatic control, thereby reducing the ability of cells to respond to neuronal activity and stress. In order to be able to utilise iPSC‐derived cells to study the early metabolic changes in AD, it is necessary to first ensure that the astrocytes behave similarly to cells ‘in vivo*’*. We and others have shown that astrocytes differentiated from iPSCs show typical stellate morphology, and express astrocyte markers including S100β, ALDH1L1 and GFAP (Oksanen et al., 2017; Shaltouki et al., 2013; Zhou et al., 2016). In the brain astrocytes breakdown glycogen under conditions of starvation in order to maintain survival of neurons, protect axons and ensure synaptic activity is maintained (Brown & Ransom, 2007). Furthermore, during neuronal activity uptake of glutamate and sodium chloride by glutamate transporters in astrocytes initiates glycogenolysis and rapid lactate production (Hertz et al., 2015), which may be used as a fuel source in neurons (astrocyte–neuron lactate shuttle), although this is still a debated area (Kann, 2023; Chih & Roberts Jr., 2003; Pellerin et al., 1998). Data presented here show that glutamate induces glycogenolysis in iPSC‐derived astrocytes and that process can be blocked using ouabain and TBOA. This demonstrates that glutamate uptake and efflux of sodium chloride is associated with glycogen breakdown. Furthermore, we demonstrate that astrocytes can store glycogen and are able to undergo glycogenolysis in response to hypoglycaemia and in response to dbcAMP. While these findings have been reported in primary rodent cells (Amaral et al., 2011; Pellerin & Magistretti, 1994; Ransom & Fern, 1997; Zwingmann & Leibfritz, 2003) and human NT2.D1 embryo carcinoma‐derived astrocytic cells (Tarczyluk et al., 2013), this is the first time that this response has been reported in human iPSC‐derived astrocytes. It is unclear why iPSC derived astrocytes did not significantly respond to isoproterenol in this study. The cells used in this study were ~45 days old and this finding may indicate that further maturation of these cells may be required.

Evidence of altered metabolic profiles in iPSC‐derived astrocytes from late‐onset AD has been previously shown (Ryu et al., 2021). Aβ is an important hallmark of AD pathology and is postulated to play a role in altered metabolism, therefore, we investigated how relatively acute treatment of Aβ altered astrocytic metabolism. iPSC astrocytes showed a concentration‐dependent pattern of reduction in glucose uptake which concurs with previous work (Abeti et al., 2011; Tarczyluk et al., 2015). The pathogenesis of amyloid‐induced reduction in glucose uptake remains a focus of research and several mechanisms have been suggested as possible pathways for glucose uptake reduction. For example, Aβ reduces glucose uptake by preventing GLUT3 fusion to the plasma membrane, despite elevated protein expression (Prapong et al., 2002). Impaired lipid peroxidation has also been proposed as a mechanism for reduced glucose uptake in AD (Robert et al., 1997). Further, the intracellular aggregation of Aβ can induce alterations in pro‐inflammatory and metabolic reprogramming that predisposes cells to die through regulated cell death pathways, a process which can induce change in glucose metabolism and mitochondrial bioenergetics (Huang et al., 2020). In this study, treatment with Aβ1‐42 oligomers induced a significant reduction in glycogen content. The mechanisms behind this amyloid‐induced reduction in glycogen turnover remain to be explained but is perhaps unsurprising since cells exhibit a reduction in glucose uptake and may therefore initiate glycogenolysis. In addition, we observed alterations in basal metabolic rates (increase oxygen consumption) and differences in glutamate uptake and lactate release following acute Aβ treatments. These data show that Aβ may interfere with glutamate uptake, which would impact one of the key roles of astrocytes in mediating neurotoxicity. This in turn would hinder lactate shuttling which is a key energy source for neurons provided by astrocytes during excitation or low glucose availability. However, further research utilising the co‐culture of neurons and astrocytes is needed to confirm dysfunction of cell–cell metabolic coupling. We demonstrate that iPSC‐derived astrocytes display features associated with a reactive state following Aβ exposure, which is in agreement with previous studies that show AD pathology in PSEN1 astrocytes (Oksanen et al., 2017). In fact, similar findings are demonstrated here, with increased Aβ generation and an increased Aβ42:40 ratio in PSEN1 astrocytes. We add further evidence of a reduction in non‐amyloidogenic AβPP processing via lower ADAM10 activity and reduced sAβPPα secretion was detected in PSEN1 astrocytes compared to controls. The relationship between the presence of the PSEN1 mutation and reduced ADAM10 activity in astrocytes is not known, and remains an interesting topic for further investigation (Elsworthy, Hill, et al., 2022).

It was hypothesised PSEN1 astrocytes which are chronically exposed to elevated, (albeit much lower) levels of Aβ, would display metabolic alterations and reactivity. Under resting conditions, we detected significantly higher oxygen consumption compared to control astrocytes, which has been previously shown (Oksanen et al., 2017; Ryu et al., 2021). Interestingly, we also demonstrate an elevation in non‐mitochondrial oxygen consumption, proton leak and a reduction in ATP‐linked oxygen consumption suggesting an alteration in the efficiency of the astrocytic aerobic system and early hypermetabolic phenotype even with a reduction in glucose uptake. This elevated metabolic profile has been previously reported in late onset AD‐derived cells (Ryu et al., 2021). Further to this, a marked elevation in maximal uncoupled aerobic capacity was detected, suggesting an adaptive response to cellular stress in the PSEN1 mutation and highlights areas for future work. PSEN1 astrocytes used in this study also demonstrated a pronounced reactive state, indicated by increased cell size, elevated inflammatory cytokine accumulation and an increase in GFAP protein levels. This supports the growing evidence of a role of reactive astrocytes in mediating aspects of AD pathology and may be a key feature of early pathogenesis (Bellaver et al., 2023; Chatterjee et al., 2021; Monterey et al., 2021). Furthermore, evidence of redox stress was identified with significantly increased lipid peroxidation, although this was not matched by any alteration in superoxide generation. The majority of lipid peroxides have been attributed to neuronal sources although evidence of astrocytic lipid peroxidation has been previously shown in in vivo AD models (Montine et al., 2002; Park et al., 2021). Metabolomic analysis of patient‐derived and Aβ‐treated astrocytes revealed significant alterations in key pathways including citric acid cycle, purine metabolism, glutamine, glutamate, arginine, alanine and aspartate metabolism. Alterations in key metabolic pathways have previously been reported in AD (Andersen et al., 2022; Ansoleaga et al., 2015; Fleszar et al., 2019; Sang et al., 2022; Zhang et al., 2021).

In this study, the healthy control astrocytes were used to compare the effects of exogenous Aβ application and the impact of PSEN1 mutations. It is important to note that the ‘healthy’ control patient used possess the APOE2/2 genotype. Recent studies have shown that that the APOE genotype significantly influences neuronal energy metabolism, with APOE4 leading to alterations in both mitochondrial and glycolytic pathways and APOE2 demonstrating the highest spare respiratory capacity (Brookhouser et al., 2021; Budny et al., 2024). Further studies have also demonstrated that APOE2 can mitigate some of the disease reputed phenotypes associated with APP processing and impacts lipid and cholesterol metabolism (de Leeuw et al., 2022; Lindner et al., 2022). These studies provide valuable insights into the potential mechanisms underlying the increased risk of AD associated with APOE and should be carefully considered in future studies.

Studying the early metabolic features of AD is crucial for developing effective treatments and understanding the disease process. Data presented herein show altered markers of AD pathology, including APP misprocessing and Aβ production, in fAD patient‐derived astrocytes. Furthermore, we demonstrate significant metabolic changes in both Aβ treated and fAD derived astrocytes, as well as elevated markers of astrocyte reactivity. Both fAD‐ and Aβ‐treated astrocytes demonstrate a bioenergetic shift to a hypermetabolic state despite a reduced uptake of glucose and glutamate. Our results highlight the impact of AD on the anaplerotic (regeneration of metabolic intermediates) and cataplerotic (loss of metabolic intermediates to provide precursors for biosynthesis) nature of astrocytes and demonstrate how the delicate balance of maintaining neurotransmitter and metabolic demands could be perturbed in response to APP misprocessing in both acute and chronic exposure to Aβ. These findings emphasise the importance of astrocytes in cerebral metabolism and highlight how this could be perturbed in neurodegeneration. Treatments targeting astrocytic reactivity as well as metabolic dysfunction may reduce or ameliorate the further development of AD and should be considered in preventative early trials in preclinical studies.

## AUTHOR CONTRIBUTIONS

**Richard J. Elsworthy:** Conceptualization; formal analysis; funding acquisition; investigation; methodology; resources; supervision; writing – original draft; writing – review and editing. **Mattea J. Finelli:** Data curation; funding acquisition; investigation; methodology; resources; software; writing – original draft. **Sarah Aqattan:** Investigation; methodology; writing – original draft. **Connor Dunleavy:** Investigation. **Marianne King:** Investigation. **Adele Ludlam:** Investigation; methodology. **Marta A. Tarczyluk:** Data curation; investigation; methodology. **Sophie L. Allen:** Investigation. **Sophie Prosser:** Investigation. **Rui Chen:** Investigation; methodology. **Sandra Martinez Jarquin:** Investigation. **Dong H. Kim:** Investigation; methodology. **James Brown:** Supervision. **Rheinhalt Parri:** Resources; supervision; writing – review and editing. **Sarah Aldred:** Funding acquisition; resources; supervision; writing – review and editing. **Eric J. Hill:** Conceptualization; funding acquisition; project administration; resources; supervision; writing – original draft; writing – review and editing.

## FUNDING INFORMATION

This work was supported by Alzheimer's Research UK [ARUK‐MID2022]. Embassy of the State of Kuwait PhD studentship funding. MJF funded by University of Nottingham Interdisciplinary Centre for Analytical Science Fund and Anne McLaren Fellowship (University of Nottingham).

## CONFLICT OF INTEREST STATEMENT

The authors declare that the research was conducted in the absence of any commercial or financial relationships that could be construed as a potential conflict of interest.

### PEER REVIEW

The peer review history for this article is available at https://www.webofscience.com/api/gateway/wos/peer‐review/10.1111/jnc.16267.

## Supporting information


Appendix S1.

## Data Availability

All data will be made freely available on reasonable request. A preprint of this article was posted on BioRxiv; 24 August 2023; https://www.biorxiv.org/content/10.1101/2023.08.23.554346v1.
