# Supplementary material for: Altered metabolic function induced by Aβ‐oligomers and PSEN1 mutations in iPSC‐derived astrocytes
Source: J Neurochem. 2024 Dec 18;169(1):e16267. doi: 10.1111/jnc.16267 (PMC11655965; doi:10.1111/jnc.16267)
Supplement: Supplementary file 1 — Appendix S1. [file JNC-169-0-s001.pdf]

SUPPLEMENTARY FILE:

Altered metabolic function induced by Aβ-oligomers and PSEN1 mutations in iPSC-derived astrocytes

Richard. J. Elsworthy[1*], Mattea. J. Finelli[2], Sarah Aqattan[5], Connor Dunleavy[1], Marianne King[3], Adele Ludlam[4], Marta A.Tarczyluk[5], Sophie L. Allen[1,6], Sophie Prosser[1], Rui Chen[7], Sandra Martinez Jarquin[7], Dong H. Kim[7] James Brown[5], H. R. Parri[4], Sarah Aldred[1], Eric J. Hill[8*].

Author affiliations:

[1]School of Sport, Exercise and Rehabilitation Sciences, College of Life and Environmental Sciences, University of Birmingham, Birmingham, UK.

[2]Biodiscovery Institute, University of Nottingham, School of Medicine, University Park Nottingham NG7 2RD

[3]**Sheffield Institute for Translational Neuroscience,** University of Sheffield, 385a Glossop Road, Sheffield, S10 2HQ

[4]School of Pharmacy, College of Health and Life Sciences, Aston University, Birmingham, B47ET

[5]School of Biology, College of Health and Life Sciences, Aston University, Birmingham, B47ET

[6]NIHR Birmingham Biomedical Research Centre, University Hospitals Birmingham NHS Foundation Trust, University of Birmingham, Birmingham, UK.

[7]Centre for Analytical Bioscience, Advanced Materials & Healthcare Technologies Division, School of Pharmacy, University of Nottingham, Nottingham, NG7 2RD

[8]Department of Chemistry, Loughborough University, Loughborough, LE11 3TU

Correspondence to: Eric J Hill

Department of Chemistry, Loughborough University, Loughborough, LE11 3TU, UK.

E-mail: e.j.hill@lboro.ac.uk

Supplementary table 1. - *Information on cell lines used for generating astrocytes from healthy control PSEN1 fAD mutation carriers.*

	**Ax0018**	**Ax0112**	**Ax0113**	**R278I**
**Diagnosis**	Healthy Control	Familial AD	Familial AD	Familial AD
**Sample type**	Dermal Fibroblast	Dermal Fibroblast	Dermal Fibroblast	Dermal Fibroblast
**Donor sex**	Male	Female	Female	Male
**Age at sampling (yrs)**	74	38	31	60
**Age of onset (yrs)**	n/a	39	45	58
**Karyotype**	Normal	Normal		
**Reprogramming method**	Episomal Vector	Episomal Vector	Episomal Vector	Episomal Vector
**Induction method**	Monolayer – Axolbio NIM [35]	Monolayer – Axolbio NIM [35]	Monolayer – Axolbio NIM [35]	Monolayer - NIM [35,79]
**Mutation**	None	PSEN1 (L286V)	PSEN1 (A246E)	PSEN1 (R278I)
**APOE status**	ε2/ ε2	ε3/ ε3	ε2/ ε3	ε2/ ε4

Note: Adapted from [34]

Supplementary table 2. - *Neural induction media (NIM) made by the addition of small molecule signalling pathway inhibitors to Essential 6 as a base medium.*

COMPONENT	AMOUNT
GIBCO™ ESSENTIAL 6™ MEDIUM (THERMOFISHER SCIENTIFIC, A1516401	50ml
XAV939 (STEM CELL TECHNOLOGIES, 72672)	10µl
LDN193189 (STEM CELL TECHNOLOGIES, 72147)	0.5µl
SB431542 (STEM CELL TECHNOLOGIES, 72232)	50µl

Note: from [34]

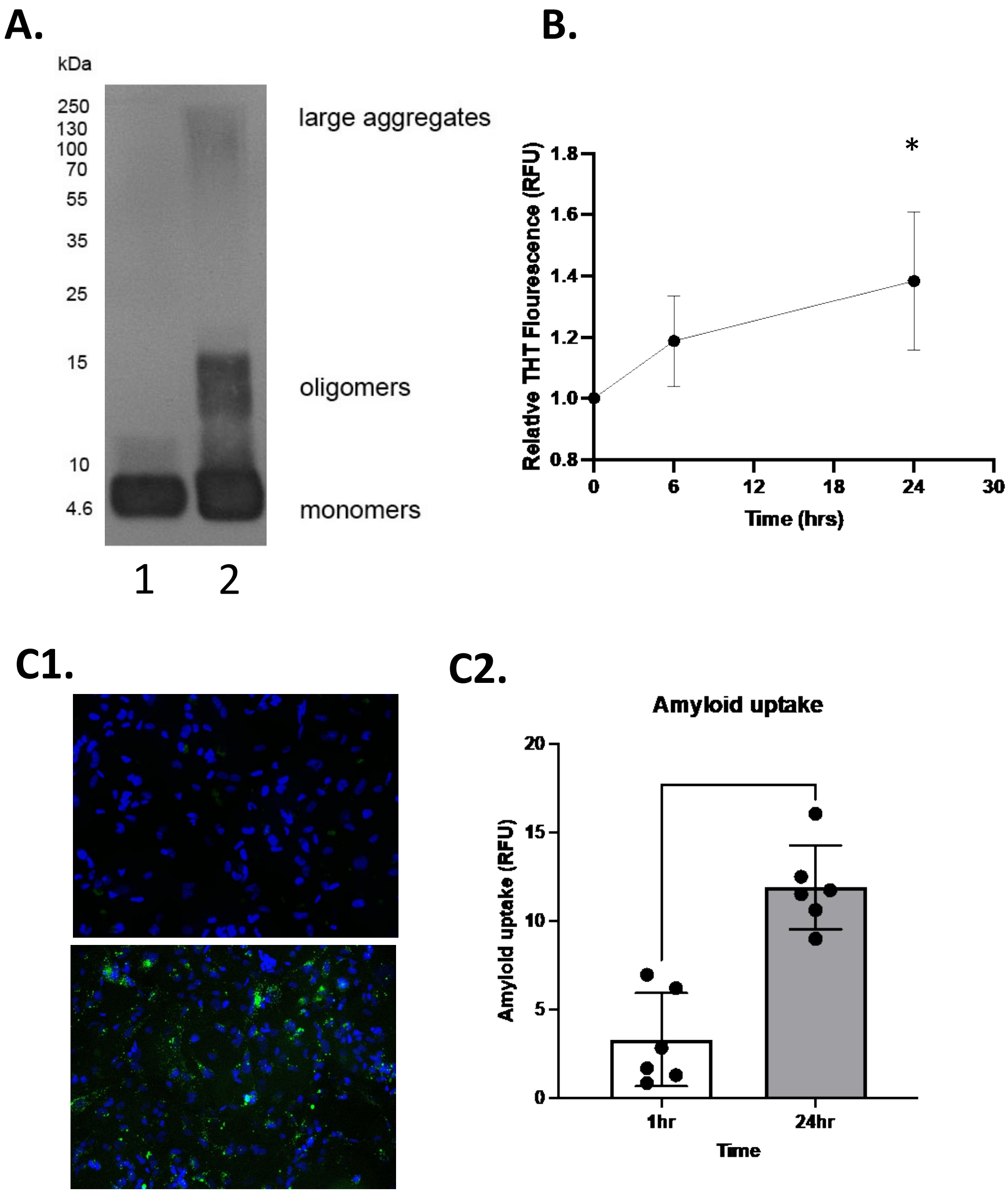

Sup. Figure 1. Preparation of Aβ1-42 oligomers. Human HFIP Aβ1-42 was resuspended in DMSO to 5mM. Monomers were diluted in F-12 culture media, without phenol red, to a concentration of 100µM and incubated for 24hrs at 4°C. Monomers (A1) and oligomers (A2) visualised using gel electrophoresis and native blotting. Increased Thioflavin-T fluorescence during oligomerization relative to 0hrs at 6hrs and 24hrs (B). Uptake of HiLytefluor-488 labelled Aβ1-42 (Anaspec 60479-01) oligomers by control astrocytes (ax0018) was seen from 1 hour and significantly elevated at 24hrs (C1 & C2). *$p<0.05$, ***$p<0.001$

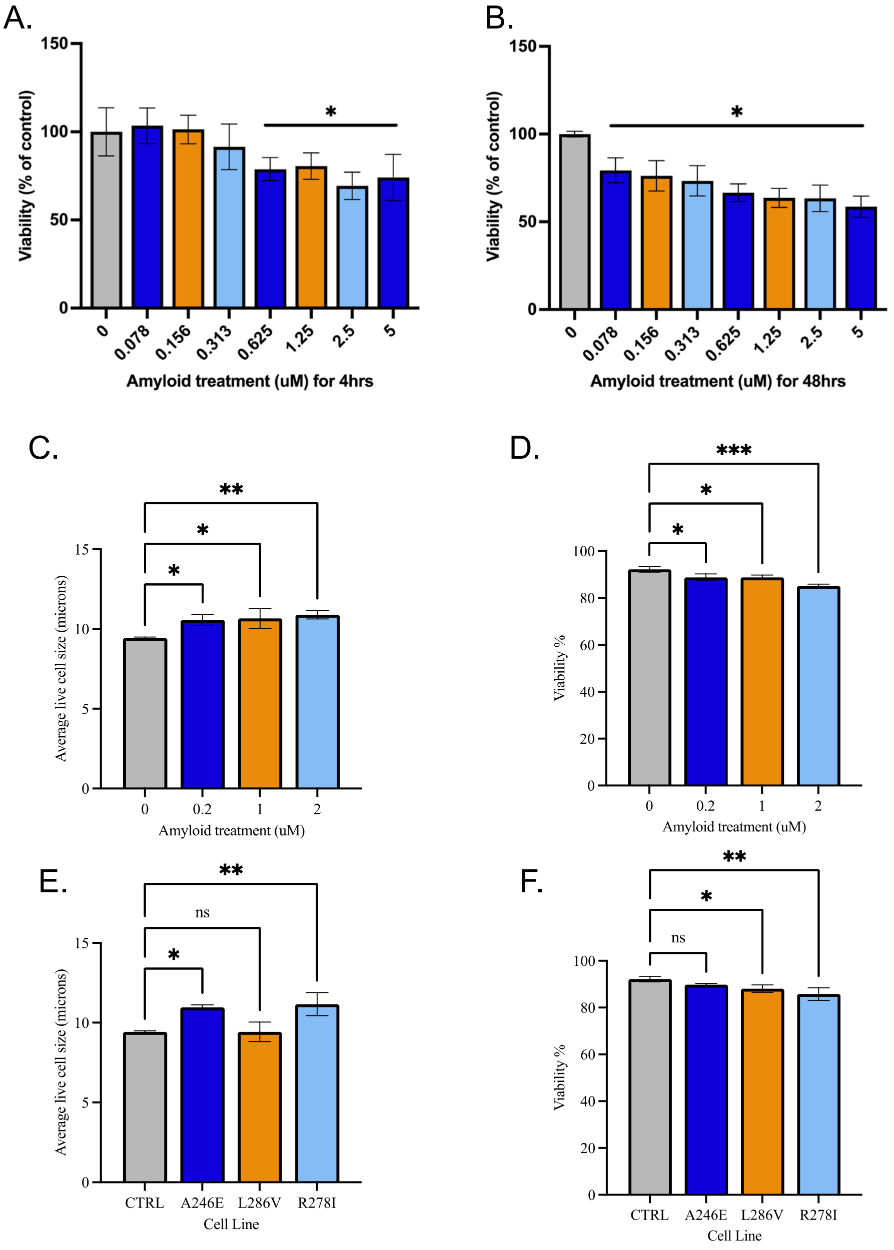

Sup. Figure 2. Summary figures showing cell viability data from MTT assay follow treatment with Amyloid beta for 4 hrs (A, left panel) and 48hr treatment (B, right panel). Cell size (microns) and percentage viability from AOPI cell counting are also shown for both amyloid beta treatments (C & D) and in PSEN1 carrying astrocytes (E & F).

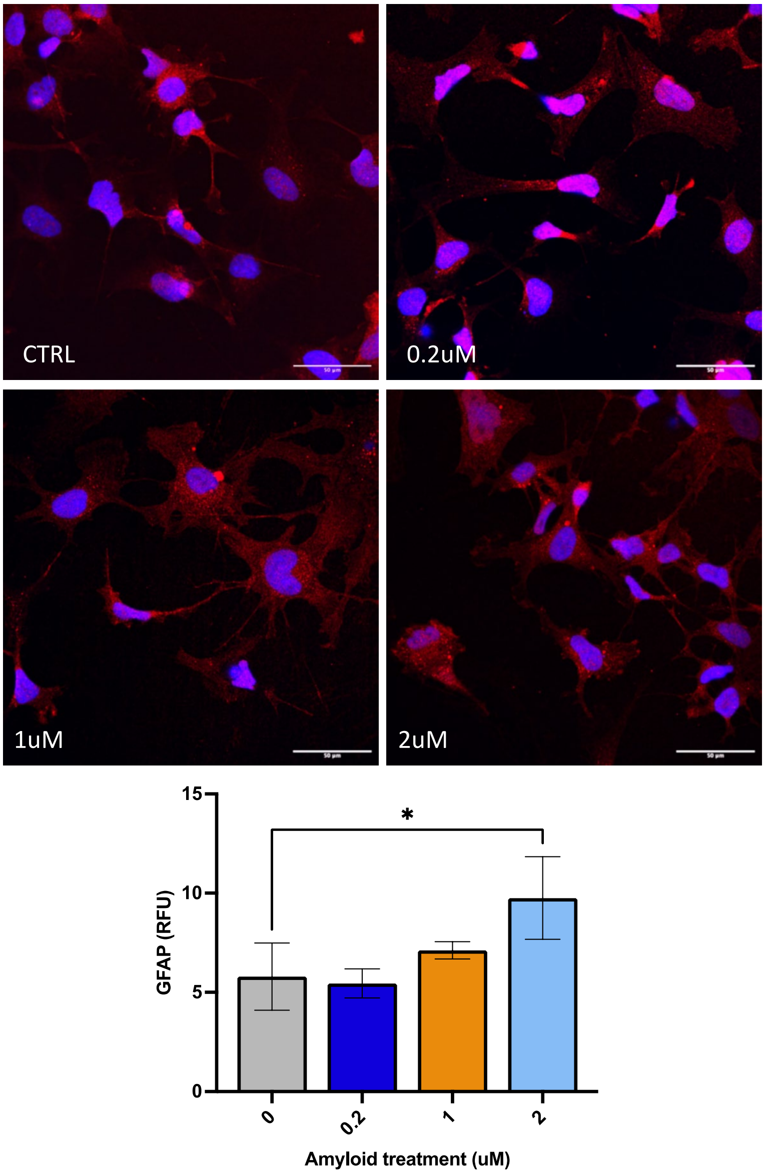

Sup. Figure 3. GFAP protein expression was quantified from ICC images taken of healthy control (AX0018, day 45) astrocytes exposed to Amyloid-beta oligomers for 48hours. Representative images are shown above with the corresponding concentration of Amyloid-beta in the bottom left corner of each image. 3 replicate treatments were imaged with 3 regions of interest averaged for each replicate. Data was analysed using a One-way ANOVA with Sidak corrections.

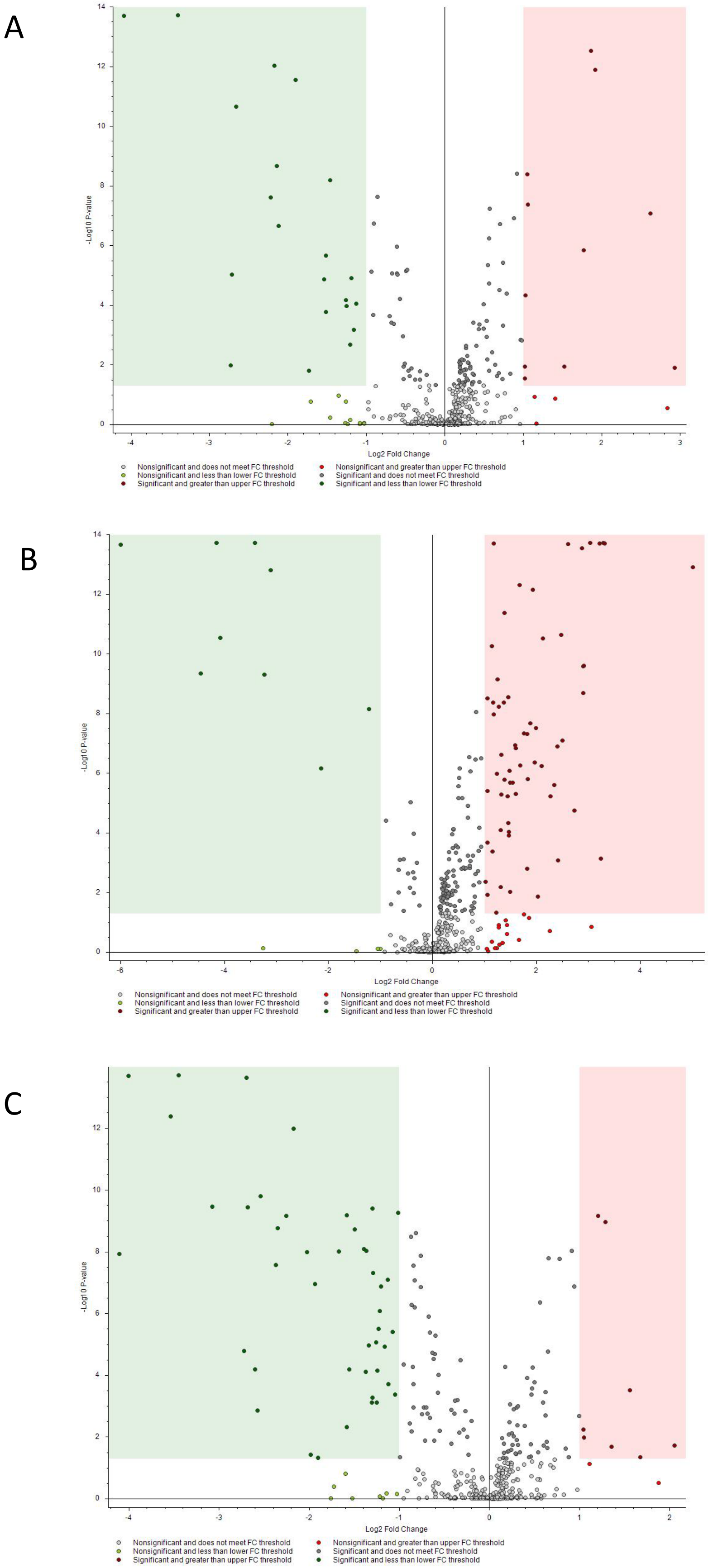

Sup. Figure 4. The volcano plots of PSEN1 astrocytes compared with control: metabolites in red were more abundant in PSEN1 astrocytes while metabolites in green were identified at higher level in control relative to PSEN1 astrocytes. (A): A246E vs. control. (B): L286V vs. control. (C): R278I vs. control.

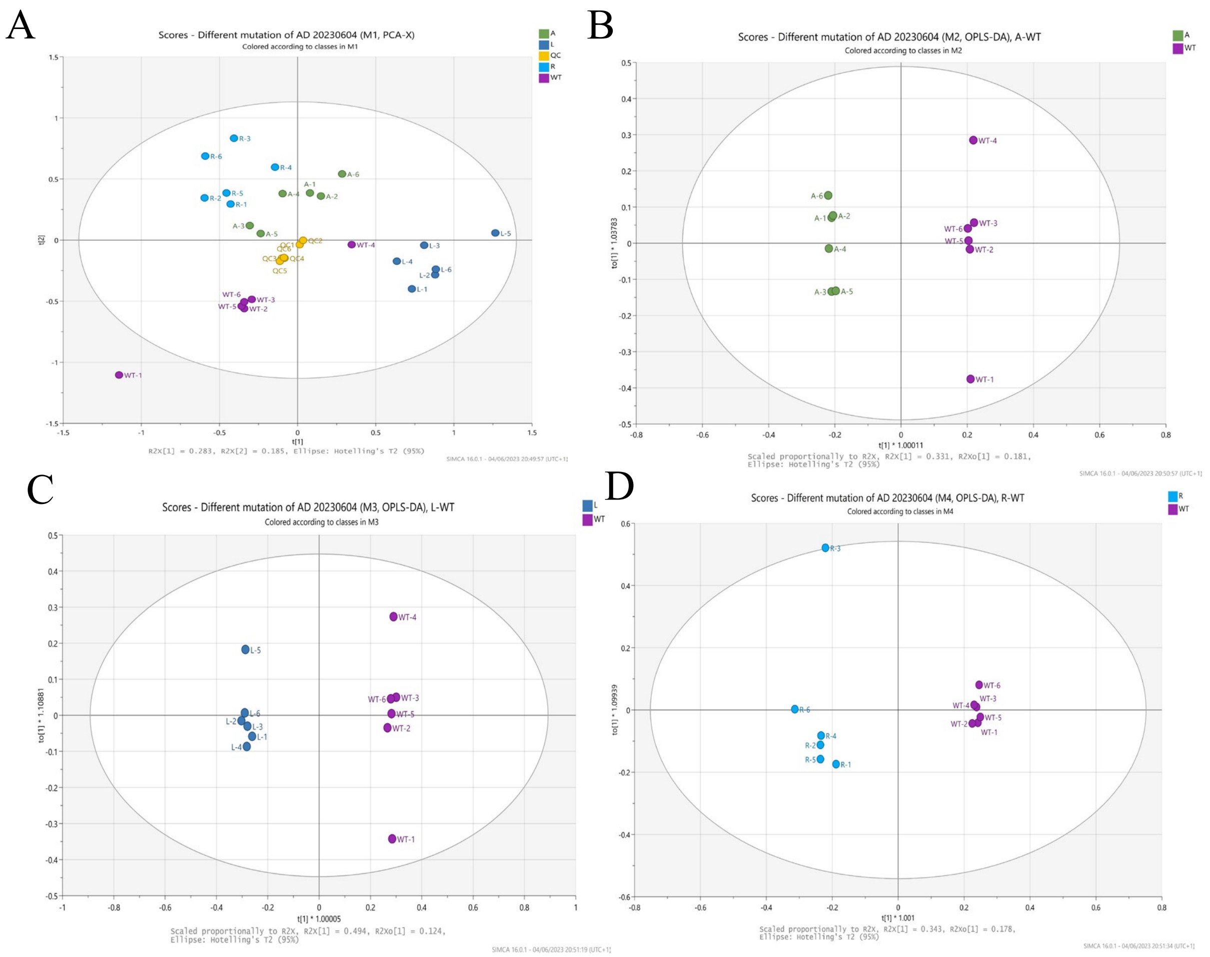

Sup. Figure 5. Summary of results of PCA and OPLS-DA results for the comparison of PSEN1 mutations with control astrocytes. (A): The PCA scores plot of different PSEN1 mutation astrocytes and control astrocytes. (B): The OPLS-DA scores plot of A264E (A) compared with control (WT): R^2Y 0.999 and Q^2 0.939. (C):The OPLS-DA scores plot of L286V compared with control: R^2Y 0.998 and Q^2 0.967. (D):The OPLS-DA scores plot of R278I mutant compared with wild type: R^2Y 0.987 and Q^2 0.931.

A

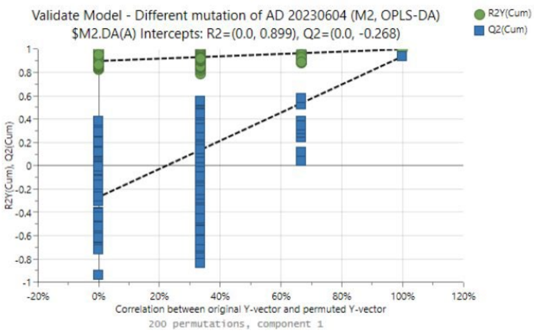

B

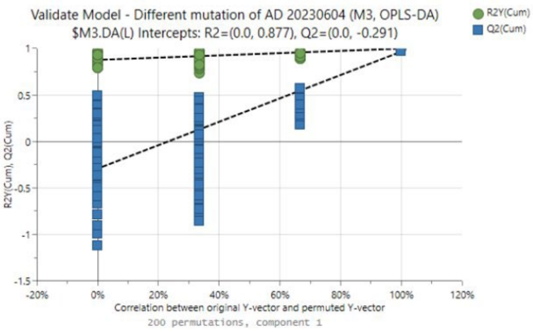

C

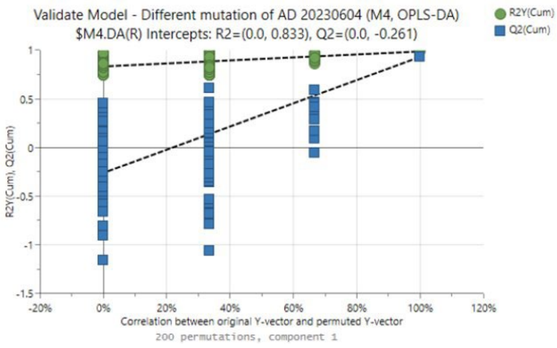

Sup. Figure 6. The permutation test results of each comparison. (A): The permutation test of A264E mutation compared with control. (B): The permutation test of L286V mutation compared with control. (C): The permutation test of R278I mutant compared with control.
